# A lightweight xAI approach to cervical cancer classification

**DOI:** 10.1007/s11517-024-03063-6

**Published:** 2024-03-20

**Authors:** Javier Civit-Masot, Francisco Luna-Perejon, Luis Muñoz-Saavedra, Manuel Domínguez-Morales, Anton Civit

**Affiliations:** 1 Robotics and Computer Technology Lab, ETSII, Universidad de Sevilla, Reina Mercedes s/n, Seville, 41018 Spain; 2 Computer Engineering Research Institute, Universidad de Sevilla, Reina Mercedes s/n, Seville, 41018 Spain

**Keywords:** Cervical cancer, Explainable AI, Deep learning, Medical imaging, Medical diagnosis aid

## Abstract

**Abstract:**

Cervical cancer is caused in the vast majority of cases by the human papilloma virus (HPV) through sexual contact and requires a specific molecular-based analysis to be detected. As an HPV vaccine is available, the incidence of cervical cancer is up to ten times higher in areas without adequate healthcare resources. In recent years, liquid cytology has been used to overcome these shortcomings and perform mass screening. In addition, classifiers based on convolutional neural networks can be developed to help pathologists diagnose the disease. However, these systems always require the final verification of a pathologist to make a final diagnosis. For this reason, explainable AI techniques are required to highlight the most significant data to the healthcare professional, as it can be used to determine the confidence in the results and the areas of the image used for classification (allowing the professional to point out the areas he/she thinks are most important and cross-check them against those detected by the system in order to create incremental learning systems). In this work, a 4-phase optimization process is used to obtain a custom deep-learning classifier for distinguishing between 4 severity classes of cervical cancer with liquid-cytology images. The final classifier obtains an accuracy over 97% for 4 classes and 100% for 2 classes with execution times under 1 s (including the final report generation). Compared to previous works, the proposed classifier obtains better accuracy results with a lower computational cost.

**Graphical abstract:**

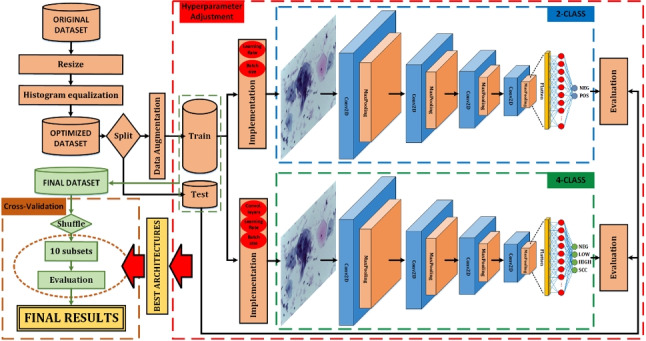

**Supplementary Information:**

The online version contains supplementary material available at 10.1007/s11517-024-03063-6.

## Introduction

Cervical cancer is a malignant tumor of the cervix, caused in 99% of cases by persistent infection with high-risk human papilloma viruses (HPV), a widespread virus transmitted by sexual contact [1]. This is mainly observed in women under 35 years of age in countries with low HPV vaccination rates and limited healthcare resources. HPV types can be classified as low risk or high risk depending on their association with benign, precancerous, or cancerous lesions.

Cervical cancer ranks fourth globally in cancers diagnosed and fourth in women’s cancer deaths, with a higher proportion of low- and middle-income countries. It is particularly present in sub-Saharan Africa [2]. In 2020, more than 600,000 new cases were diagnosed worldwide (6.5% of all female cancers) and more than 340,000 deaths related to cervical cancer were reported. In general, 84% of new cases and around 90% of deaths occur in these countries [3]. Furthermore, in high-income countries, this incidence is 7–10 times lower.

Early detection of cervical cancer plays a key role in treatment and can significantly reduce the risk of death; therefore, specialized laboratory equipment and pathologists are required to perform these tests. An interview with medical specialists (cardiologists and oncologists) in the United States and Germany in 2016 summarized that 66% of the critical decisions they made were based on the results obtained by the pathology and laboratory medicine (PALM) services [4]. This implies that access to high-quality and timely PALM services is needed to support health care systems.

However, for example, in sub-Saharan Africa, the number of pathologists is approximately one per million patients, a ratio approximately 50 times lower than that of high-income countries (such as the United States or the United Kingdom) [5]. Due to this situation, it is essential to look for diagnostic aid systems to reduce pathologist workload and, as a result, to allow them to increase the number and quality of their diagnostic decisions.

The usefulness of diagnostic aid systems is not to replace the pathologist’s work, but to provide a tool for the pathologist to allow rapid initial screening of samples and to present a detailed report that helps the pathologist with his or her decision.

At this point, the application of artificial intelligence (AI) techniques is of great importance in the design of classifier systems capable of extracting characteristics from images and differentiating between those that indicate some type of disease and those that represent a healthy patient [6].

The application of this type of technique in medical imaging provides three main benefits:

- Mass case screening: easily diagnosable cases may have a quick analysis, reducing the time spent by the specialist.
- Specialists’ workload reduction: as a consequence of the above, the specialist can spend more time on severe and/or difficult-to-diagnose cases. As a secondary implication, false negatives could be reduced.
- Diagnosis time reduction: as a consequence of the previous benefits, both the specialist and the patient could know the diagnosis in a shorter time, and, therefore, the action plan in case of detection of the disease could be streamlined, improving the survival rates in many cases.

As a result, numerous AI-based diagnostic aids have been developed in recent years. These systems have the advantage of providing fast classification but require a sufficiently large dataset so that, during the training phase of these tools, the most relevant features can be correctly extracted from the data provided by each sample.

The use of AI techniques can be seen in the field of healthcare in multiple aspects: from the analysis of physiological signals [7–9] to the study of bad habits and abnormalities during activities of daily living [10–13]. Moreover, in relation to this work, the use of techniques derived from AI and machine learning (ML) is widely extended in the field of medical image analysis, making use of advanced deep learning (DL) techniques based on convolutional neural networks (CNN). These techniques have been applied in multiple research works in recent years, obtaining very positive results with a correct diagnosis rate higher than 80% [14–16]; even reaching, in several cases, accuracy values above 95% [17–19].

However, errors obtained in this type of diagnostic aid system must be minimized because their results are critical, and diagnosis failure can even be fatal. Therefore, the analysis of errors, as well as disturbances caused by their variations, is of great importance in this type of system [20, 21]. Hence, such a classifier must follow a thorough optimization process that includes the analysis of variations in the input (by applying techniques that randomly select the sets of images to be used in the tests). Mechanisms such as hold-out or cross-validation are commonly used to improve the robustness of the developed system.

However, very few diagnostic aid systems achieve a 100% accuracy, because those systems are usually tested on a subset of samples from the dataset itself used to train them (sharing similarities in many cases) [22–24]. Thus, in future classifications, some errors may occur if samples from other medical centers are evaluated or different scanning devices are used (among other possible variables involved) [25].

This fact leads to a certain mistrust of automated diagnostic support systems among healthcare professionals. Moreover, another problem with DL systems is the fact that, when they are trained, the weights of the neural network connections do not provide information understandable by the user that helps him/her to know the objective criteria used to perform the classification. For this reason, these systems are known as “black boxes” [26].

Due to this, in recent years, the use of explainable artificial intelligence (xAI) and explainable deep learning (xDL) technologies has gained very significant importance. These technologies, through various and varied subsequent analyses, provide information on the objective classification criteria used in the automatic system [27, 28]. This objective information obtained after these analyses is of great importance, not only to detect possible classification errors but also to allow the healthcare professional to understand the decisions made in the correct classifications. This is the reason why this type of analysis is essential in medical diagnostic aid systems [29, 30].

The authors’ research group has extensive experience in the field of machine learning and deep learning applied to e-Health. This experience can be appreciated, for example, in the field of physiological signal monitoring and/or processing [31], biomechanical gait studies [32], fall detectors [33], etc. Moreover, in addition to this, the group provides experience in the field of medical imaging processing using convolutional neural networks (CNN), having developed multiple aid systems for the diagnosis of cancer and other diseases [24, 34].

**Fig. 1:**
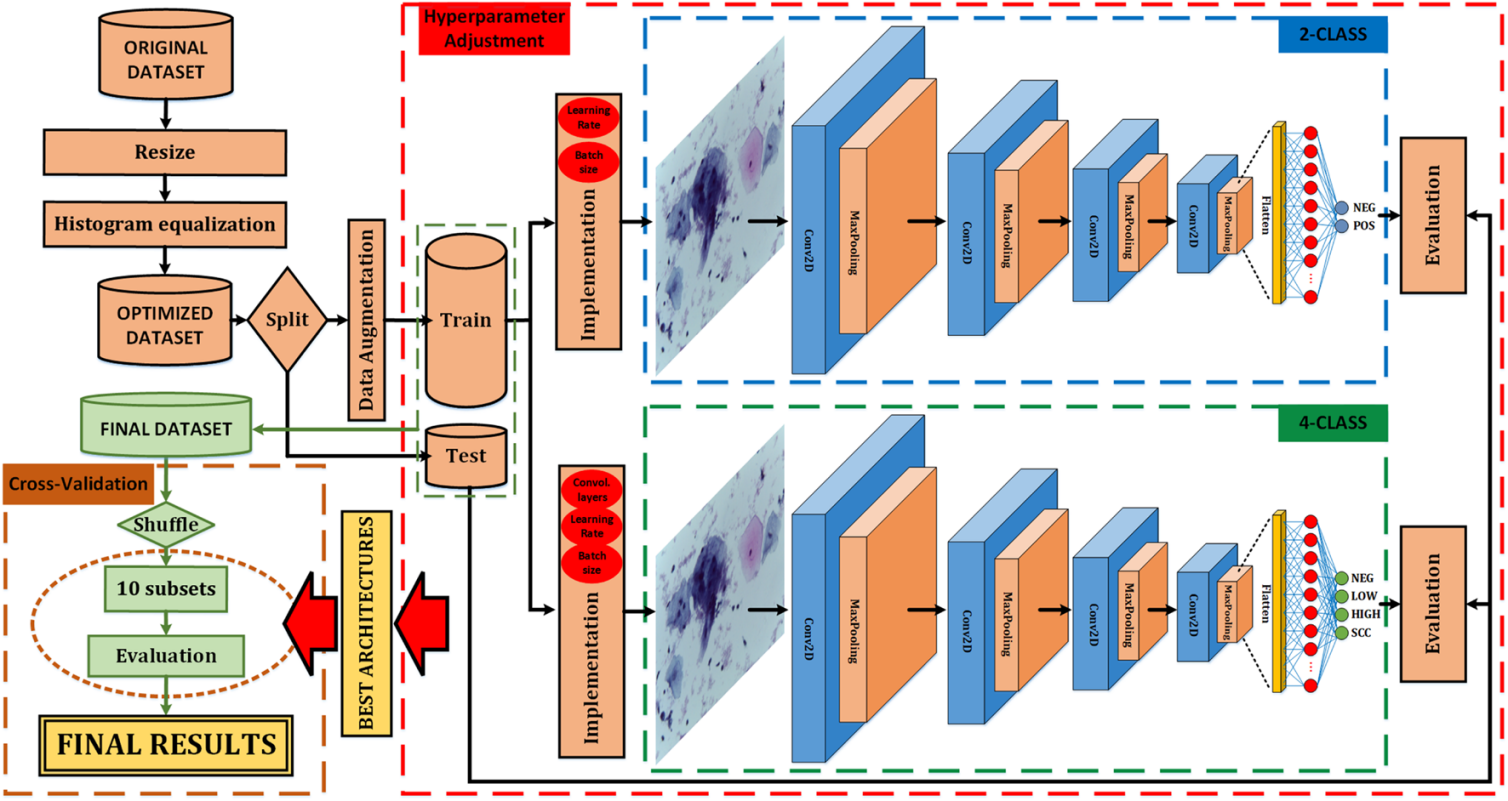
Work’s progress summary

So, in this work, a diagnostic aid system for precancerous and cervical cancer lesions is designed, implemented, and tested using liquid-based cytology imaging. In addition to explaining the procedure and the results obtained, a comparison will be made with previous work in the area. And finally, to provide better information to the healthcare professional, xDL techniques will be used to study the areas of the images in which the classifier has focused to perform decisions and to provide a detailed report to the healthcare professional regarding the confidence level of the system’s decision for each class. In this way, not only the possible diagnosis result but also the areas of images that the classifier has mainly used to obtain that diagnosis can be provided to the pathologist (together with a detailed classification report about the confidence of belonging to each class). So, the highlights of this work are as follows:

- A better optimization process based on a 4-stage analysis.
- Better results compared to previous work.
- Lighter classifier for mass screening and for use in low-resource countries as it can be implemented in computers with lower processing power or cloud-based, associated processing costs are reduced.
- High detailed reports thanks to xAI techniques.

The rest of the manuscript is organized as follows: In Sect. 2, the description of the developed classifier, the metrics used to evaluate it, the description of the xDL techniques performed, and a search for recent similar works are detailed. The results obtained after testing the classifier and the explainable deep learning reports are explained in Sect. 3, together with a comparison with previous work. Finally, Sect. 4 draws the discussion and conclusions of this work and proposes future research lines.

## Materials and methods

This section presents tools and systems designed to detect cervical cancer in liquid cytology samples. To do so, we will first detail the dataset used, its structure, and the preprocessing applied to it. Then, the characteristics of the classifiers developed will be presented, as well as the justification for their choice. Finally, we will explain how these classifiers have been evaluated and what information is provided to the pathologist.

In summary, the graphical abstract of the work presented can be seen in Fig. 1.

**Table 1:** Original dataset distribution and class renaming for this work

Original class	Renamed class	Number of images
NILM	NEG	613
LSIL	LOW	163
HSIL	HIGH	113
SCC	SCC	74

### Dataset

The dataset used in this work consists of a total of 963 images subdivided into four subsets, representing the four classes of precancerous and cancerous lesions of cervical cancer according to the standards under The Bethesda System. This dataset is known as the “Mendeley liquid cytology dataset” and was shared under the Creative Commons license in 2020 [35]. According to the authors, the pap smear images were captured at $\times 40$ magnification using the Leica ICC50 HD microscope, which were collected and prepared using the liquid-based cytology technique from 460 patients. The full content of this dataset is shown in Table 1.

**Fig. 2:**
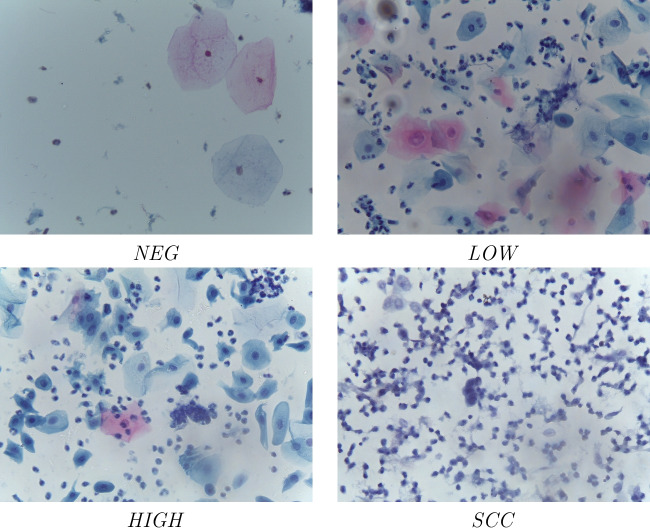
Example of images from the dataset

The acronyms used in the dataset are the following: NILM for “Negative for Intraepithelial malignancy” (renamed “NEG” in this work), LSIL for “low squamous intraepithelial lesion” (renamed “LOW” in this work), HSIL for “high squamous intraepithelial lesion” (renamed “HIGH” in this work), and SCC for “squamous cell carcinoma” (this acronym is retained in this work). An example of each class type is shown in Fig. 2.

The main problem found in this dataset is the class balance: a DL classifier tries to maximize the number of samples it classifies during its training process. Therefore, in the case of this dataset, it will try to maximize the NEG class. However, in this work, we are trying to correctly classify all classes, so we will have to apply pre-processing techniques to the dataset to improve the distribution of images of each class. These techniques are described in the following.

### Preprocessing

The preprocessing process applied to the dataset consists of three steps: resizing, equalization, and data augmentation (in this order).

First, the images are resized from $2048 \times 1536\, \text {to}\,\, 240 \times 180$ pixels to reduce the computational load of the classifier and speed up the training and classification processes. After that, a histogram equalization is performed on the reduced images.

And, finally, the data augmentation process is performed. Due to its complexity, this step is justified and detailed next.

As indicated above, the dataset has a significant imbalance problem. The most populated class has almost 9 times the number of images of the least populated class. To solve this problem, data augmentation techniques are applied on the dataset to increase the number of images of the least populated classes. However, the least populated class (SCC) has only 74 images, and applying data augmentation techniques on it to reach the number of images of the most populated class (NEG with 613 images) would imply obtaining more than 8 artificial images for every real image. If this process were carried out, it could condition the results of the classifier, as the augmented images have characteristics similar to the original ones, and this fact would hinder the classifier.

This is the reason why, in order to balance the dataset, it was decided to use only 150 images for each class. In this way, only one augmented image would be needed for each real image of the SCC class, and one augmented image per each 3 real images of the HIGH class. To obtain the new images, a random rotation (90, 180, or 270 degrees) or a zoom modification (between 5 and 15%) is applied offline to both classes (SCC and HIGH), until the number of 150 images for these classes is reached.

**Table 2:** Dataset distribution after data augmentation (D.A.) for each subset and class

Class	Training subset		Validation subset		Testing subset	
	*Before D.A.*	*After D.A.*	*Before D.A.*	*After D.A.*	*Before D.A.*	*After D.A.*
NEG	105	105	15	15	30	30
LOW	105	105	15	15	30	30
HIGH	68	105	15	15	30	30
SCC	29	105	15	15	30	30

As can be observed, the augmented images from HIGH and SCC classes are located in the training subset

**Fig. 3:**
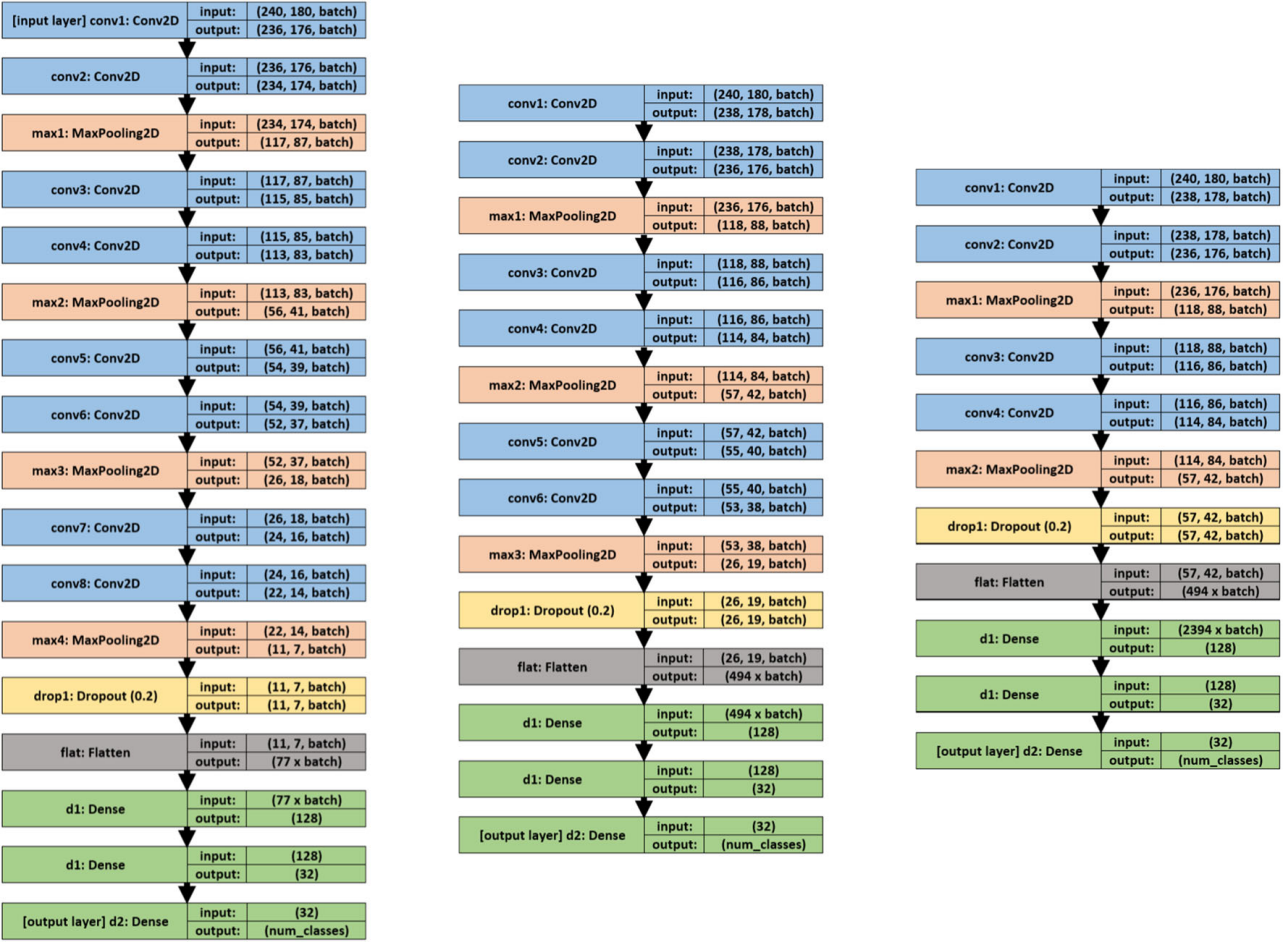
Classifiers tested: (left) 8 convolutions + 4 max polling; (middle) 6 convolutions + 3 max polling; (right) 4 convolutions + 2 max polling

With respect to the NEG and LOW classes, where the number of required images (150 per class) is exceeded, a random selection process of 150 images is performed online (at the time of loading the dataset), so that all images are involved in the training processes and tests performed (although only a subset of 150 per class in each test).

For each class, a 70% of the images is used for training (105), a 10% for validation (15), and a 20% for test (30). Regarding the augmented images of the SCC and HSIL classes, it is important to note that, in the subset splitting process, they are forced to be placed in the training subset. In this way, for the validation and test processes, we work with original images and not with augmented ones. The final distribution can be seen in Table 2.

### Classification system

In this work, multiple classifiers based on convolutional neural networks (CNN) are evaluated considering the number of convolutional layers involved and the values of their hyperparameters (learning rate and batch size). The neural network architectures used are completely customized (without using pre-trained models); so, we use combinations of the basic layers: convolutional layers, dense layers, polling layers, dropout layers, and flatten layers. A summary of the networks used is shown in Fig. 3.

In addition, to be able to compare the results of this work with previous works, classifiers are not only designed and tested for the four classes indicated above but also implemented and tested using only two classes (negative and positive) as many previous works use this approach. In the two-class classifiers, the dataset is divided into negative images (entirely from the NEG class) and positive images (by combining LOW, HIGH, and SCC classes in equal proportions).

For all these classifiers, multiple tests have been carried out varying the values of the hyperparameters (learning rate and batch size). The process of finding the best classifier is divided into four phases:

- Phase 1: coarse-grained adjustment. In this first step, 16 batch sizes (even values from 2 to 32 inclusive) and 8 learning rates (1e-5, 5e-5, 1e-4, 5e-4, 1e-3, 5e-3, 1e-2, and 5e-2) are evaluated over the three different classifier architectures. This has required 384 training processes, using 100 epochs for each.
- Phase 2: Selection of the best candidates. Taking into account the accuracy and loss results of each individual training, the best combination of batch size and learning rate for each architecture is selected, resulting in 3 candidate networks. The best candidates are named CNN1 (8-convolution classifier), CNN2 (6-convolution classifier), and CNN3 (4-convolution classifier) (see Fig. 3).
- Phase 3: fine-grained adjustment. The three candidate networks obtained from the previous phase are trained with 1000 epochs each. In addition, to guarantee the results and reduce the randomness generated by the selection of the training, validation, and test subsets, the cross-validation technique is applied to the complete dataset, obtaining ten non-coincident combinations of the subsets. In this way, each network architecture is trained and evaluated 10 times with 1000 epochs. The accuracy deviation in these tests provides a measure of the robustness of the model independently of the dataset division, thus ensuring that the choice of architecture and parameters is not due to the specific dataset split.
- Phase 4: Selection of a winner. Taking into account the results obtained from the above cross-validation process, the mean, standard deviation, and median values are extracted. The architecture with the best mean value is considered the winner (in the case of similar values, the one with the smallest standard deviation will be selected). The results used for this final comparison are those obtained from the mean values of the previous phase.

### Evaluation

To evaluate the effectiveness of the classification system, well-known metrics are used: accuracy (most used metric), sensitivity (also known as recall), specificity, precision, and F1_score_ [36]. To this end, the classification results obtained for each class are tagged as “true positive” (TP), “true negative” (TN), “false positive” (FP), or “false negative” (FN). According to them, the high-level metrics are presented in the following equations:

1 $$\begin{aligned} Accuracy= & {} \sum _{c}\frac{TP_c \!+ TN_c}{TP_c +\! FP_c +\! TN_c + FN_c}, c \!\in \! classes\nonumber \\ \end{aligned}$$

2 $$\begin{aligned} Specificity= & {} \sum _{c}\frac{TN_c}{TN_c + FP_c}, c \in classes\end{aligned}$$

3 $$\begin{aligned} Precision= & {} \sum _{c}\frac{TP_c}{TP_c + FP_c}, c \in classes\end{aligned}$$

4 $$\begin{aligned} Sensitivity= & {} \sum _{c}\frac{TP_c}{TP_c + FN_c}, c \in classes\end{aligned}$$

5 $$\begin{aligned} F1_{score}= & {} 2*\frac{precision * sensitivity}{precision + sensitivity}. \end{aligned}$$

About those metrics:

- Accuracy: all samples classified correctly compared to all samples (see Eq. 1).
- Specificity: proportion of “true negative” values in all cases that do not belong to this class (see Eq. 2).
- Precision: proportion of “true positive” values in all cases that have been classified as it (see Eq. 3).
- Sensitivity (or Recall): proportion of “true positive” values in all cases that belong to this class (see Eq. 4).
- F1_score_: It considers both the precision and the sensitivity (recall) of the test to compute the score. It is the harmonic mean of both parameters (see Eq. 5).

These metrics are common to all ML/DL systems, but there are other metrics commonly used in medical diagnostic systems; this is the case of the ROC curve (receiver operating characteristic) [37], because it is the visual representation of the true positives rate (TPR) versus the false positives rate (FPR) as the discrimination threshold is varied. Usually, when the ROC curve is used, the area under the curve (AUC) is used as a value of the system’s goodness of fit.

Therefore, the classifier systems developed in this work will be evaluated according to all the metrics detailed in this subsection.

### Report generation

As commented before, the use of the Grad-CAM algorithm for CNN-based systems is very widespread. Moreover, it can be adapted to classification problems (as is the case in this work), visual question answering, and captioning. It uses the gradients of any target concept, flowing into the final convolutional layer, to produce a coarse localization map that highlights the important regions in the image for predicting the concept.

**Fig. 4:**
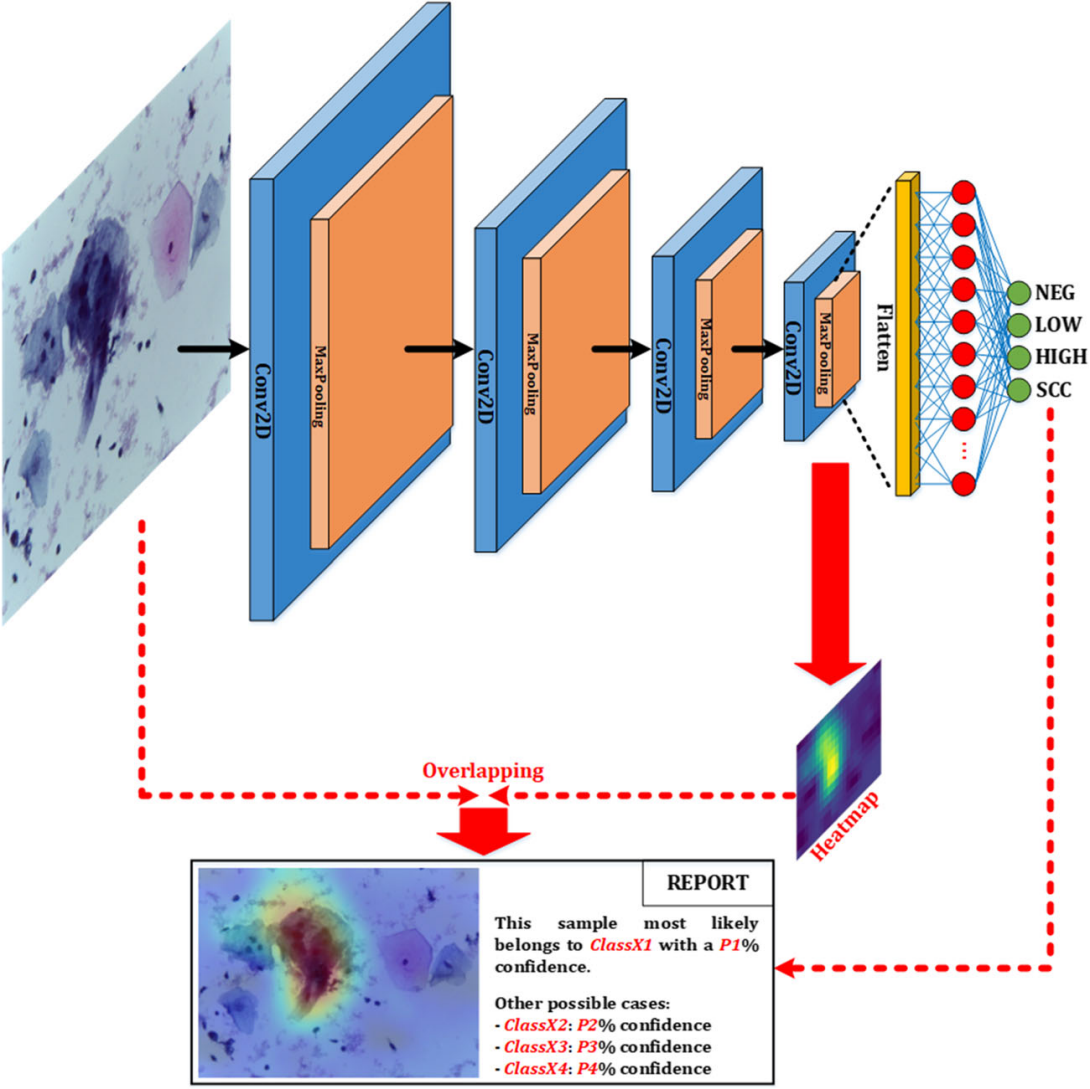
System’s final report given to the healthcare professional

Therefore, after the classification performed by the diagnostic aid system developed in this work, the information provided to the healthcare professional is completed with an explanation of the decision taken based on the use of the Grad-CAM algorithm on the evaluated image and a text report about the confidence of the system regarding each of the output classes (obtained from the values obtained previously to the softmax activation of the last layer). A summary of the final report generation process is shown in Fig. 4.

Based on this detailed report, the healthcare professional can make the final decision, which could be to validate the proposed results or to carry out a more detailed human-based study of the sample.

In the “Results” section, the Grad-CAM algorithm is applied to several testing images, providing the final report for all cases. Before this, it is important to carry out a search for similar works, to be able to compare them with this work at the end of the manuscript.

### Previous works

A global search is performed in the main search engines (Scopus, IEEExplorer, and Google Scholar) with the following keywords: “cervical cancer,” “cytology,” and “deep learning.” The results obtained are filtered by year, restricting the works to those published in the last 5 years. Preprints or arXiv/bioRxiv works waiting for acceptance are not selected.

An initial filtering of works that use liquid cytology images has not been considered appropriate, as this type of imaging has been widely used only relatively recently. Due to this and to the datasets used by each study, different nomenclatures can be found in the classifications made by those works. Table 3 shows the equivalence of the nomenclature between the cancer and precancer classification systems [38].

**Table 3:** Cervical cancer and pre-cancer: terminology for cytological and histological reporting

Cytological classification		Histological classification	
Pap	Bethesda	CIN	WHO
Class I	Normal	Normal	Normal
Class II	ASCUS / ASC-H	Atypia	Atypia
Class III	LSIL	CIN1	Koilocytosis
Class III	HSIL	CIN2	Moderate dysplasia
Class III	HSIL	CIN3	Severe dysplasia
Class IV	HSIL	CIN3	Carcinoma in situ
Class V	Carcinoma	Carcinoma	Carcinoma

The most common nomenclatures used in publicly available datasets are the Bethesda System and the CIN (cervical intraepithelial neoplasia) classification. This last one evolved in 1968, to take into account the different natural histories seen with different degrees of dysplasia (CIN1/mild, CIN2/moderate, CIN3/severe). On the other hand, the Bethesda system was developed in the 1990s at the US National Cancer Institute, distinguishing between high-grade squamous intraepithelial lesions (HSIL, which combines CIN2 and CIN3) and low-grade squamous intraepithelial lesions (LSIL, equivalent to CIN1). Furthermore, in the 2001 Bethesda System, atypical cells are divided into ASCUS (atypical squamous cells of undetermined significance) and ASC-H (atypical squamous cells: cannot exclude a high-grade squamous intraepithelial lesion), but not all public datasets include this final classification.

The results after the searching process and the elimination of articles not focused on the design of a classifier reflect a total of 23 works. The selected works are presented below with a brief summary.

- Xu et al. [39]: 2-class classifier using colposcopy images with random forest (RF), k-nearest neighbors (kNN), logistic regression (LR), AdaBoost, support vector machine (SVM), and a pre-trained CNN model.
- Elakkiya et al. [40]: 2-class classifier using colposcopy images with custom CNN.
- Alyafeai et al. [41]: 2-class classifier using colposcopy images with pre-trained CNN models.
- Jeftic et al. [42]: 2-class classifier using liquid cytology images with statistical mechanisms.
- Sanyal et al. [43, 44]: 2-class classifier using liquid cytology images with custom CNN classifier.
- Teramoto et al. [45–47]: 2-class classifiers using liquid cytology images with pre-trained CNN models.
- Mulmule et al. [48], Nagadeepa et al. [49]: 2-class classifiers using liquid cytology images with statistical mechanisms and custom CNN models.
- Kanavati et al. [50]: 2-class classifier using liquid cytology images with an ensemble composed by a pre-trained CNN model and an SVM.
- Isidoro et al. [51]: 2-class and 3-class classifiers using liquid cytology images with SVM classifier.
- Manna et al. [52]: 2-class and 4-class classifiers using colposcopy images with pre-trained CNN models.
- Zhu et al. [53]: 2-class and 5-class classifiers using liquid cytology images with pre-trained CNN models.
- Huang et al. [54]: 3-class classifier using liquid cytology images with statistical mechanisms.
- Zhang et al. [55]: 3-class classifier using colposcopy images with custom CNN.
- Zhang et al. [56]: 4-class classifier using colposcopy images with pre-trained CNN models.
- Hussain et al. [57], Martinez-Más et al. [58], Kundu et al. [59]: 4-class classifiers using liquid cytology images with pre-trained CNN models.
- Kuko et al. [60]: 5-class using liquid cytology images with custom CNN.
- Nambu et al. [61]: 6-class classifier using liquid cytology images with pre-trained CNN models.

**Table 4:** Best results obtained for each architecture after all the combinations of learning rate and batch size hyperparameters

Architecture	Learning rate	Batch size	Accuracy	Loss
8 Convolutions	1e−4	24	98.95	0.03
	1e−4	28	97.56	0.12
	5e−4	32	96.89	0.10
6 Convolutions	5e−5	12	96.70	0.14
	5e−4	22	96.79	0.15
	5e−4	30	97.89	0.10
4 Convolutions	1e−5	30	97.20	0.16
	5e−5	24	97.44	0.19
	1e−4	20	98.46	0.09

As can be seen, there is no standard classifier used. Most commonly, mechanisms based on convolutional neural networks (CNN) are found, but there are also cases where more statistical methods and ensemble combinations are used. Regarding the classified classes, many works opt for defining a binary state, but it depends on the dataset used and the nomenclature used. The most commonly used nomenclatures are the Bethesda System (up to five classes) and the CIN classification (up to six classes).

If we make a preliminary comparison, it can be observed that all of them try to optimize the classification accuracy (instead of the computational requirements); and, moreover, the vast majority of them do not present a detailed final report. These two points will be taken into account in the work presented in this manuscript.

## Results

In this section, the implementation, optimization, and evaluation process of the classifiers presented previously is detailed and discussed. We will start with the 4-class classifier, and, after that, the same process will be followed for the binary classifier.

**Fig. 5:**
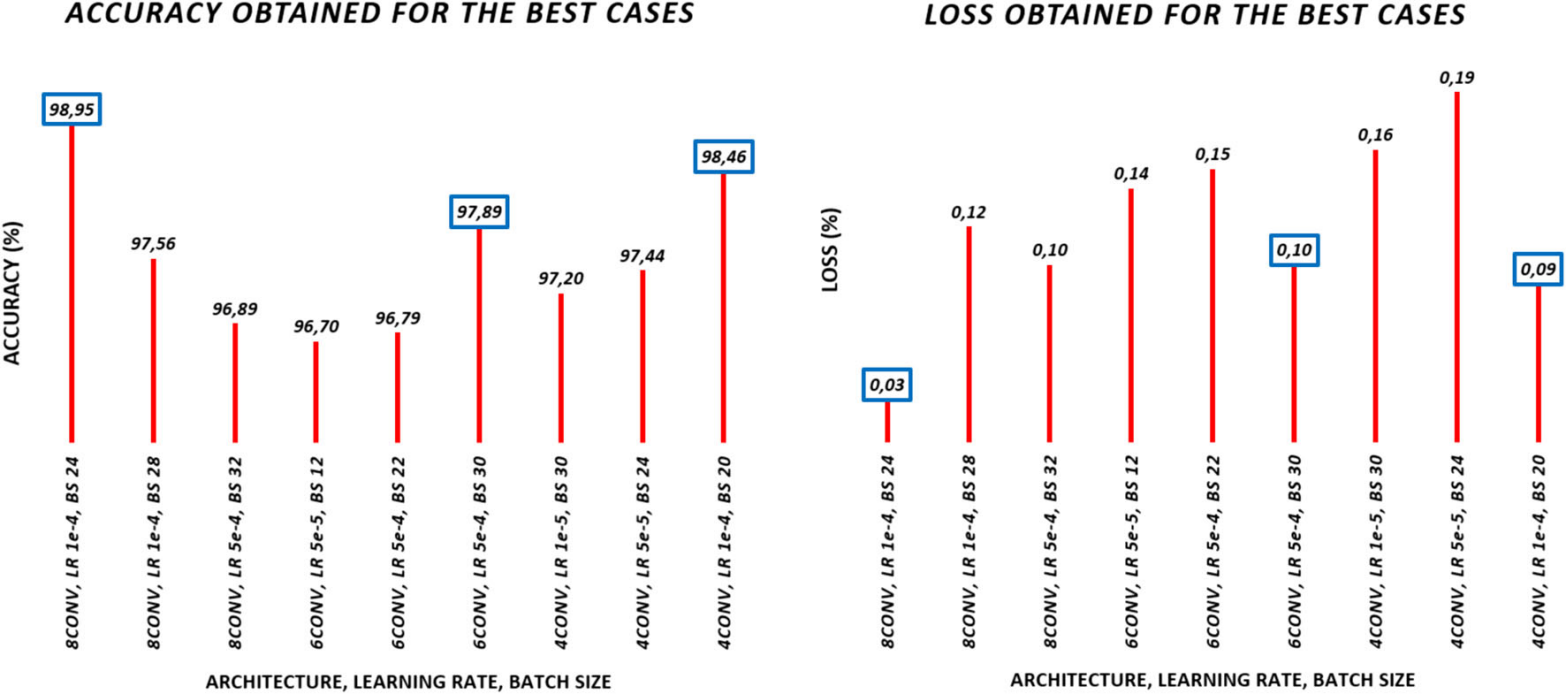
Accuracy and loss summary for the best cases of the three architectures

### 4-class classifier

#### Phase 1

Short training processes (100 epochs) are performed on the three convolutional networks detailed above, varying the learning rate and the batch size. Eight different learning rates and sixteen batch sizes are used; this implies a total of 128 training processes for each network (384 training processes in total). The selection of training, validation, and test subsets is randomized at the beginning of each training, taking into account that the augmented images are located only in the training subset. The results obtained for each case are evaluated by the test accuracy and loss for the three CNNs, and the best results obtained are shown in Table 4. The results obtained for all the cases can be accessed in the Supplementary Material document.

The cases in which the trained systems do not obtain acceptable classification rates are for low values of the learning rate with low values of batch size. If we take these premises into account, several tests may be avoided; however, these first results will help to filter the most promising cases, which will be used in later phases.

For all the results obtained, higher learning rate values prevent the systems from converging correctly to an optimal solution. This happens in all three networks for the cases 5e-3, 1e-2, and 5e-2. Similarly, at lower values of the learning rate, low values of batch size also prevent the convergence of the system (as is the case for batch sizes below 12). In general, the best learning rates for all networks are 5e-5, 1e-4, and 5e-4, with the best results obtained in general for the case of 1e-4 (although, exceptionally, the 6-convolution network shows its maximum with 5e-4). Regarding the batch size, it can be seen that, in general, the best results are obtained for values between 20 and 30, although other good results are obtained for lower values, so we may think that this parameter is not restrictive.

It is important to note that the five best cases for each convolutional network are marked in blue in the Supplementary Material document. However, those values will be analyzed in the next phase to obtain the best candidate from each convolutional network.

#### Phase 2

Best cases from the previous phase are plotted graphically, where the highest peaks (in the case of accuracy) and the deepest valleys (in the case of loss) can be observed. This information is presented in Fig. 5.

The first three values of each image refer to the best cases of the first architecture (8 convolutions), the next three are those of the second architecture (6 convolutions), and the last three are those of the third architecture (4 convolutions).

**Table 5:** Three final candidates obtained at the end of phase two

Name	CNN model	Learning rate	Batch size
CNN1	8conv+4max+3dense	1e−4	24
CNN2	6conv+3max+3dense	5e−4	30
CNN3	4conv+2max+3dense	1e−4	20

For each of these blocks, we will determine the best case, which will be used in the next phase.

If we look at the three best cases of the first architecture, there is a clear winner with a 98.95% accuracy; although there is only a difference of 1.4% between it with the second classifier. Both have a learning rate of 1e-4 and similar batch sizes: 24 and 28. However, if we look at the error obtained in the validation, the case with the batch size of 24 improves significantly, reducing the loss value to a 25% (0.03) compared to the other case.

Considering now the three best cases of the second architecture, there is one candidate that stands out from the others. It corresponds to a learning rate of 5e-4 and batch size 30, and obtains an accuracy value of 97.89%. Moreover, if we look at the error obtained, it is also the candidate with the lowest loss value with 0.10.

And finally, for the three best cases of the third architecture, there is also a clear winner with a 98.46% accuracy and a loss value of 0.09.

**Table 6:** Fine-grained study with the best candidates obtained from the previous phase: [CNN1] 8 conv., lr 1e-4, bs 24; [CNN2] 6 conv., lr 5e-4, bs 30; [CNN3] 4 conv., lr 1e-4, bs 20

	subset1	subset2	subset3	subset4	subset5	subset6	subset7	subset8	subset9	subset10	TOTAL		
	Acc	Acc	Acc	Acc	Acc	Acc	Acc	Acc	Acc	Acc	Mean	STD	Median
CNN1	99.95	93.75	94.93	99.87	97.97	99.87	97.50	98.00	96.93	97.75			
CNN2	97.64	95.00	94.74	96.90	92.40	96.90	96.05	97.19	93.22	94.35	95.44	1.50	95.00 (S.2)
CNN3	94.94	91.00	96.04	88.74	95.04	96.90	92.82	87.22	96.03	91.80	93.05	2.74	92.82 (S.7)

Based on these findings, Table 5 shows a summary of the three final candidates and the nomenclature used hereafter to name them.

#### Phase 3

Now, the three candidates are compared. For this purpose, the following two actions are performed:

- First, the original dataset is randomly split offline into 10 nonmatching datasets (each with a train subset, a validation subset, and a test subset according to the split detailed in the previous section). A cross-validation process will be performed on these ten datasets, obtaining in each case the accuracy, and the best candidate from the training set will be selected.
- Secondly, for the training of this phase, the learning rate and batch size characteristics of each candidate will be taken into account, and the training time will be extended up to 1000 epochs. This is intended to allow all networks to achieve the best possible convergence (which was not always the case with 100 epochs, especially in the eight-convolution network).

The results obtained with the 10 cross-validation subsets for the three candidates are presented with the test accuracies in Table 6.

Table 6 does not only show the accuracy results for the 10 cross-validation subsets by each candidate but also three more columns where a summary of the results obtained by each candidate is presented. In this final summary, the mean accuracy value is detailed with its standard deviation (these final columns form part of the next-phase analysis).

Looking at the individual results for each of the training sessions, candidate CNN1 outperforms the other two in 8 of the 10 cross-validation training sessions. In the two cases in which it is not the winner, candidate CNN1 comes second (being bettered in one case by candidate CNN2 and by candidate CNN3 in the second case).

At first glance, the CNN1 candidate appears to be the best overall performer (looking for the mean accuracy and the mean loss); however, an evaluation of the computational requirements of the three systems is performed to decide the best final candidate.

In this study, the three candidate architectures are retrained with their learning rate and batch size. And, for each case, the average realized execution time for each classification is noted. These tests are run on two different hardware architectures: the first one has a powerful graphics card (GeForce GTX 1080Ti), and the second one runs the classifications using only CPU processing.

The results are shown in Table 7. However, in the Supplementary Material document, a larger table is included where the execution times for these candidates with a variable batch size between 1 and 50 can be observed.

**Table 7:** Execution time study performed for CNN1, CNN2, and CNN3 using two hardware platforms: only an Intel i7 CPU, and adding a GeForce 1080Ti GPU

Candidate	GPU (GeForce GTX 1080Ti)	CPU (Intel i7-10700K @ 3.8GHz)
CNN1	0.0112±0.0063	0.2083±0.0071
CNN2	0.0140±0.0084	0.2546±0.0060
CNN3	0.0042±0.0040	0.1116±0.0035

Time indicated in seconds

In the final stage of the optimization process, these data will be analyzed in detail before deciding on the final classifier.

#### Phase 4

If we look closely at the results of the previous phase (shown in the last three columns of Table 6), we can see that the CNN1 candidate has an average accuracy value that is more than 2% higher than the second-place candidate (CNN2); moreover, in terms of standard deviation, we can see that the CNN1 candidate has a better result too. In any case, the CNN3 candidate is completely discarded, as the values obtained are very distant from the other two (both in terms of accuracy and standard deviation).

Regarding the execution times, the CNN3 results are the lowest (as may be expected due to the low complexity of the network), although the poor classification accuracy (compared to the others) makes us discard this option. Taking into account the absolute execution times obtained by the other two candidates (Table 7), CNN1 classifier is faster than CNN2 using GPU (0.0112 vs 0.0140 s) and without GPU (0.2083 vs 0.2546 s). Therefore, CNN1 achieves better classification results and better execution times than CNN2, although the differences are not very significant.

**Table 8:** Final results obtained for each classification class with CNN1 and CNN2

Candidate	Class	Accuracy	Specificity	Precision	Sensitivity	F1_score_
CNN1	neg	100	100	100	100	100
	low	100	100	100	100	100
	high	97.50	98.89	96.55	93.33	94.91
	scc	97.50	97.78	93.55	96.67	95.08
	TOTAL	97.50	99.17	97.50	97.50	97.50
CNN2	neg	100	100	100	100	100
	low	99.17	98.89	96.77	100	98.36
	high	95.83	97.78	93.10	90.00	91.52
	scc	95.00	96.67	90.00	90.00	90.00
	TOTAL	95.00	98.33	95.00	95.00	95.00

**Fig. 6:**
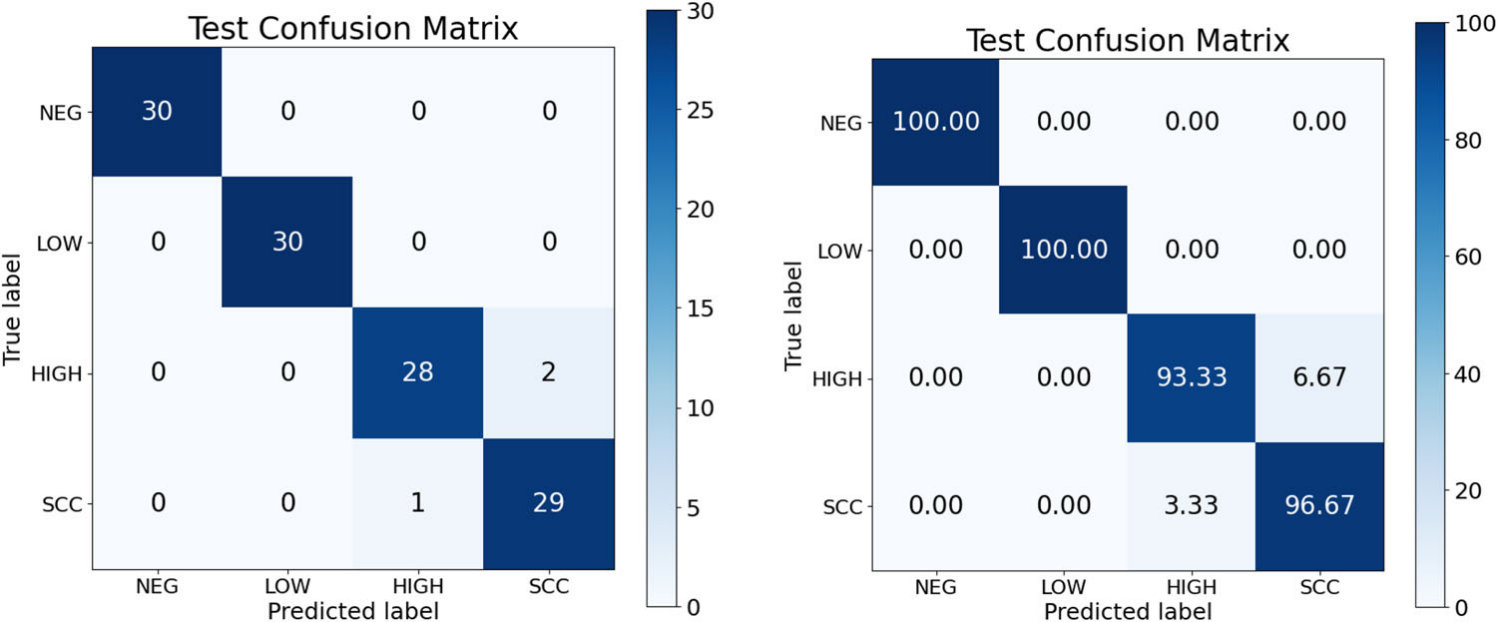
Test Confusion Matrix for the 4-class winner classifier (CNN1): (left) absolute values; (right) percentage values

Therefore, although candidate CNN1 could be directly selected as the best option for the classification system, it is important to analyze the reason for this difference in accuracy.

To do so, the results provided by both candidates are studied in detail. However, because ten different training sessions were conducted with the different cross-validation subsets for each candidate, there is some doubt regarding which of these training processes best reflects the final result of each candidate. For this purpose, we use those trained systems whose accuracy value corresponds to the median of the ten ranking results shown in Table 6.

So, looking at the last column of Table 6, the value of the accuracy median is reflected and, in parentheses, the subset that corresponds to that accuracy value is shown. Thus, the model obtained after training the candidate with this specific subset will be the one selected as the final model for each candidate: therefore, the model trained with subset 7 will be used for candidate CNN1 and the model trained with subset 2 will be used for candidate CNN2.

Taking these premises into account, the results obtained for each class independently can be seen in Table 8 for both CNN1 and CNN2.

Although both candidates exhibit a very good behavior for NEG and LOW classes, CNN2 obtains false positive cases, which are the most dangerous in this case. Moreover, CNN2 candidate achieves worse ranking results for HIGH and SCC classes. Therefore, the candidate finally selected as the final model for the classifier is CNN1 (8 convolutions, 4 maxpolling layers, and 3 dense layers; trained with a learning rate of 1e-4 and a batch size of 24; with the CNN weights obtained from the resulting model trained with subset 7).

The CNN1 confusion matrix is presented in Fig. 6.

The final results show that the classifier system implemented in this work obtains very high results (almost 98% accuracy) when classifying the samples of the four classes; showing a perfect behavior for the NEG and LOW classes. For the HIGH and SCC classes, it shows a low failure rate (around 5%). Even so, these failures are classified as a high or very high-risk class, which means that the patient will need to see a doctor for further testing. Not surprisingly, the most critical cases in this type of classifier system are those in which a sample from a sick patient is classified as healthy; but such cases do not occur in this system.

**Fig. 7:**
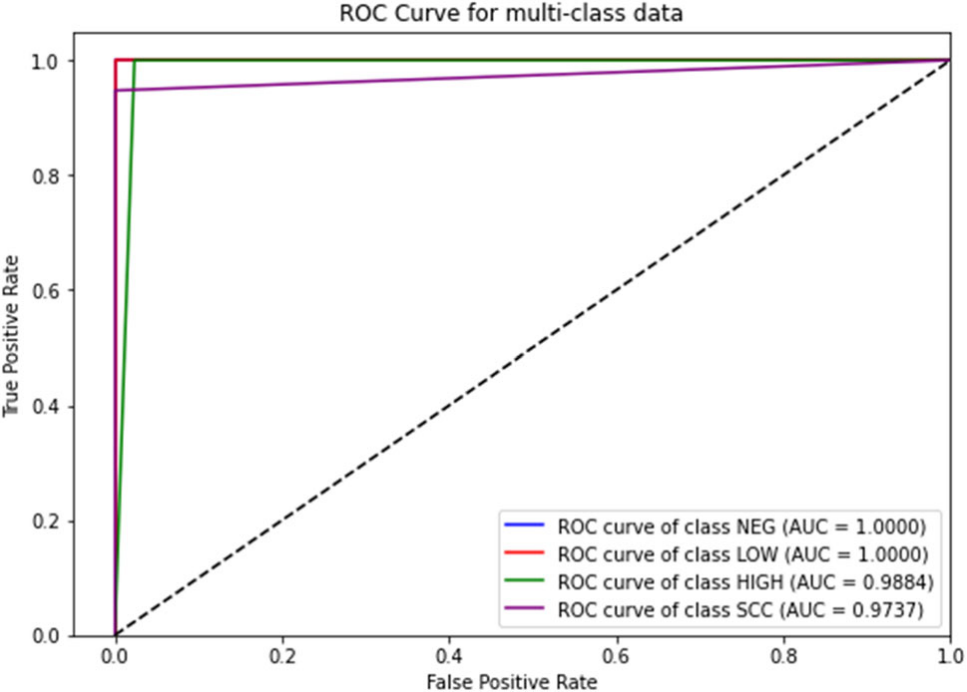
ROC curves for the four classes of CNN1 classifier (with individual AUC values)

Finally, a receiver operating characteristic (ROC) curve analysis was performed to assess the diagnostic accuracy of each class at each temporal resolution, with special emphasis on the area under the curve (AUC), which is a commonly used metric in this type of diagnostic aid system. The results can be observed in Fig. 7.

In Fig. 7, it can be observed that the NEG and LOW classes have an AUC of 100%. For the other classes, HIGH has an AUC of almost 99% and SCC has an AUC above 97%.

In these studies, AUC results are considered excellent for AUC values between 0.9 and 1, good for AUC values between 0.8 and 0.9, fair for AUC values between 0.7 and 0.8, poor for AUC values between 0.6 and 0.7 and failed for AUC values between 0.5 and 0.6. [62, 63]. Thus, the results obtained are excellent for all classes.

### Two-class classifier

For this classifier, a positive case will be equivalent to any nonnegative state. Therefore, a negative case will be the only one in which no abnormality is detected in the tissue.

The NEG class of this classifier shall have the same distribution and content as the NEG class of the 4-class classifier. However, for the case of the POS class, a mixture of samples of the three classes LSIL, HSIL, and SCC from the previous case is made. This mixture is completely equal for each of the various subsets (training, validation, and test), obtaining the subsets for training (35$\times$3 images), validation (5$\times$3 images), and testing (10$\times$3 images). On this occasion, the process of searching for the optimal classifier is simplified, reducing the range of batch size (16, 20, 24, 28, and 32) and learning rate values (1e-5, 1e-4, and 1e-3).

After 45 training sessions, it is observed that the best results are obtained with the 8-convolution architecture (with a significant deterioration in the other architectures). And, among the 15 systems trained for this architecture, the one that obtains the best results is the one trained with a learning rate of 1e-4 and batch size 20. For this case, we obtain 100% accuracy for the test set and a loss value of 0.02. All results for the 8-convolution architecture are shown in the Supplementary Material document.

As expected, the results for all metrics are 100%, as well as the confusion matrix and the ROC curve. Therefore, the corresponding tables and figures (which can be accessed in the Supplementary Material document) are omitted in the manuscript.

At this point, the different alternatives for the design of both classifiers have been explained, showing the final architectures chosen and the classification results for each one of them. In the remainder of this section, the results obtained by both classifiers will be used for comparison with the previous work detailed in Sect. 2.

### Works comparison

Following the guidelines indicated in Sect. 2, a search has been done for cervical cancer classification systems using deep learning techniques. It is important to note that, within the range of years used for the search (2017 to 2021), the publicly available works that meet all the requirements indicated previously are mainly concentrated in recent years (2019, 2020, and 2021). Some works related to cervical cancer detection were conducted in the first years and are more focused on medical innovations for treatment and sociological studies than on the creation of classifiers. However, those works focused on classifiers are included in this study as well. Moreover, the works with the best results have been found in the last years.

Therefore, taking into account the restrictions provided and the above explanation, nineteen works have been selected: one published in 2017, five published in 2019, eight published in 2020, and five published in 2021.

The summary of the selected work with its main attributes and results is shown in Table 9.

**Table 9:** Comparison with works published in recent years on neural network-based classifiers for cervical cancer and pre-cancerous lesions

Work	Images	Classifiers	Classes	Evaluation results (%)
Xu, T. [39]	Colposcopy	CNN (CaffeNet), RF	[2] negative, positive	Acc: [*CNN*] 78.4, [*SVM*] 77.2
		GBDT, AdaBoost, SVM		AUC: 82.3, 84.6, 83.3, 84
		MLP, LR, kNN		82.5, 79.7, 81.4, 73.5
Zhang, X. [55]	Colposcopy	Custom CNN	[3] type1, type2	Acc: 80.1
			type3 (no normal)	
Jeftic, B. [42]	Liquid cytology	RF, GBM, AdaBoost	[2] normal, cancer	Sen: 79, 77, 75, 76, 77, 72
		SVM, XGBoost, GLMNet		Spe: 81, 84, 76, 81, 81, 87
				AUC: 88, 86, 85, 85, 87, 86
Sanyal, P. [43]	Liquid cytology	Custom CNN	[2] normal, abnormal	Acc: 94.75; Sen: 95.63
		(4 CL, 4 ML, 3 DL)		Spe: 79.85
Teramoto, A. [45]	Liquid cytology	CNN (VGG-16)	[2] benign, malignant	Acc: 79.2; Sen: 89.3
				Spe: 83.3; AUC: 93.2
Sornapudi, S. [46]	Liquid cytology	CNNs: ResNet-50	[2] normal, abnormal	Acc: 78.18
		VGG-19, DenseNet-121		Pre: 89.13, 88.23, 91.52, 72.30
		Inception-v3		Sen: 88.18, 89.70, 80.15, 91.36
Alyafeai, Z. [41]	Colposcopy	CNN (GoogleNet)	[2] benign, cancer	Acc: 68.24; Sen: 59.70
				Spe: 77.43; AUC: 82
Isidoro, D. [51]	Liquid cytology	SVM	[2] normal, abnormal	[2] Pre: 89.7; Sen: 91.7; Spe: 88.3; F1: 90.7
			[3] normal, light (LSIL)	[3] Pre: 85.1; Sen: 95.4; Spe: 77.8; F1: 89.9
			high (HSIL and SCC)	
Sanyal, P. [44]	Liquid cytology	Custom CNN	[2] normal, abnormal	Acc: 95.46; Sen: 94.28; Spe: 96.01
		(5 CL, 5 ML, 3 DL)		
Zhang, T. [56]	Colposcopy	CNN (DenseNet), SVM	[4] normal, CIN1	[*SVM*] Acc: 63.27; Sen: 38.46; Spe: 71.85
			CIN2, CIN3+	[*CNN*] Acc: 73.08; Sen: 57.56; Spe: 78.55
				AUC: 76.3
Hussain, E. [57]	Liquid cytology	CNNs: AlexNet, VGG-16	[4] normal, LSIL	Acc: 82, 87.16, 85.16
		VGG-19, ResNet-50	HSIL, SCC	91.78, 92.61, 95.12
		ResNet-101, GoogleNet		
Kuko, M. [60]	Liquid cytology	Custom CNN	[4] normal, ASCUS/ASC-H	Acc: 90.37; Sen: 96.33; Spe: 83.59
		(3 CL, 3 ML, 2 DL)	LSIL, HSIL	
Bao, H. [47]	Liquid cytology	CNN (VGG-16)	[2] normal, abnormal	Acc: 95; Sen: 93; AUC: 76.2, 75.5
Martínez-Más, J. [58]	Liquid cytology	CNN (AlexNet)	[4] normal, ASCUS	Acc: 88.8; Sen: 92; Spe: 83
			LSIL, HSIL	
Elakkiya, R. [40]	Colposcopy	Custom CNN	[2] normal, abnormal	[2] Acc: 98.55; Pre: 98.66; Sen: 100
		(GAN)	(later, 3 subtypes)	(values obtained from training set)
Manna, A. [52]	Colposcopy	3-CNN ensemble	[2] normal, abnormal	*Inception-v3 + Xception + DenseNet-201*
		Inception-v3, Xception	[4] normal, LSIL	[2] Acc: 98.55
		DenseNet-121, DenseNet-201	HSIL, SCC	[4] Acc: 99.23
		DenseNet-169, ResNet-50		(values obtained from training set)
		ResNet-101, VGG-16, VGG-19		
Zhu, X. [53]	Liquid cytology	XGBoost	[2] negative, positive	[2] Acc: 95.21; Sen: 83.96
			[4] ASCUS/ASC-H	Spe: 94.64; AUC: 96.73
			LSIL, HSIL, SCC	[5] Acc: 80.43
Kundu, R. [59]	Liquid cytology	CNN ensembles	[4] normal, LSIL	*No ensemble (Inception-v3):*
		Inception-v3, ResNet-34	HSIL, SCC	[4] Acc: 97.9; Pre: 97.9; Sen: 97.5; F1: 97.7
		DenseNet-161	[5] superficial, parabasal	[5] Acc: 88.2; Pre: 88.6; Sen: 89.9; F1: 89.1
			metaplastic, koilocytotic	*Best ensemble: Inception-v3 + DenseNet-161:*
			dyskeratotic	[4] Acc: 99.5; Pre: 99.1; Sen: 98.9; F1: 98.9
				[5] Acc: 96.3; Pre: 95.5; Sen: 96.3; F1: 95.8
Nambu, Y. [61]	Liquid cytology	CNN (ResNeSt)	[5] normal, ASCUS/ASC-H	Acc: 90.5; F1: 70.5
			LSIL, HSIL, SCC	
Kanavati, F. [50]	Liquid cytology	CNN + RNN	[2] NIML, Neoplastic	AUC: 89%-96%
Huang, P. [54]	Liquid cytology	SVM, DT, kNN	[3] LSIL, HSIL	[*MLP*] Acc: 94.25%
			Normal	[*DT*] Acc: 82.20%
				[*kNN*] Acc: 77.16%
Mulmule, P. [48]	Liquid cytology	ANN, SVM, RF	[2] Cancer, Normal	[*MLP*] Acc: 96.4%
				[*SVM*] Acc: 82.6%
				[*RF*] Acc: 90.6%
Nagadeepa, C. [49]	Liquid cytology	SVM, CNN, RF, MLP	[2] Cancer, Normal	[*SVM*] Acc: 97%
				[*CNN*] Acc: 95.3%
				[*RF*] Acc: 94%
				[*MLP*] Acc: 95.2%
This work	Liquid cytology	Custom CNN	[2] negative, positive	[2] Acc: 100; Pre: 100; Sen: 100; Spe: 100
		(8 CL, 4 ML, 3 DL)	[4] normal, LSIL	F1: 100; AUC: 100
			HSIL, SCC	[4] Acc: 97.5; Pre: 95.00; Sen: 95.00; Spe: 98.33
				F1: 95.00; AUC: 100, 100, 98.84, 97.37

The evaluation metrics presented are related to the test subset results (unless otherwise specified)

**Legend:**

*CL* convolutional layers                   *ML* maxpolling layers                               *DL* dense layers

*RF* random forest                          *GBDT* gradient-boosted decision tree       *SVM* support vector machine             *DT* decision tree

*MLP* multilayer perceptron              *LR* logistic regression                               *kNN* k-nearest neighbors                 *RNN* recurrent neural network

*GBM* gradient-boosted machine       *GAN* generative adversarial network

It can be seen that most of the selected works use CNNs in their classifier. However, the main differences are centered on the classes detected by each classifier. As in this work two different classifiers are developed and evaluated (2-class and 4-class classifiers), the comparison will be divided into two parts: on the one hand, works that implement 2-class classifiers; and, on the other hand, works that classify more than 2 classes.

**Table 10:** 2-class classifiers complexity regarding the number of layers used in the CNN

Work	Model	N_CL_	N_ML_	N_DL_
Sanyal et al. [43]	Custom CNN			
Sanyal et al. [44]	Custom CNN	5	5	
Bao et al. [47]	VGG-16	13	5	
Elakkiya et al. [40]	Fast RCNN + GAN	40+	10+	5
Manna et al. [52]	Ensemble (Inception-v3 + Xception + DenseNet-201)	60+	20+	10+
This work	Custom CNN	8		

In the case of works that use more than one model, the less-complex one is indicated; but, in the case of works that use ensembles, the summation of the layers is shown

In general, the classifiers developed in this work perform better than the classifiers implemented in previous work. There is no 2-class classifier that improves the one presented in this work; while, for the 4-class classifier, there are a couple of previous works whose results are very similar to this one (even better): the exhaustive comparison that follows will show the differences between them and why, from the computational point of view, the classifier developed in this work is more efficient.

It should be noted that some of the works cited (6 out of 19) do not balance the training dataset (or at least they do not discuss this topic). Thus, they may present biased results. This is the case for works: Zhang et al. [55], Jeftic et al. [42], Sanyal et al. [44], Martínez-Más et al. [58], Elakkiya et al. [40], and Kundu et al. [59]. Moreover, other works give the results obtained from the training set (not the test one), like Elakkiya et al. [40], Manna et al. [52]; so, the test results may be lower than those shown in Table 9.

Other particular characteristics are detailed below:

- Sanyal et al. [43]: better results were obtained with a 10-epoch training than for other cases with more training epochs. Due to the lack of a dropout layer, it seems that a phenomenon of overfitting is taking place for this classifier.
- Teramoto et al. [45]: This work does not include a dropout layer either.
- Hussain et al. [57]: This work collects the dataset used in our work (Mendeley liquid-based cytology dataset).
- Kuko et al. [60]: 19.18% of the misclassified samples are abnormal cells classified as normal. These are the cases that any diagnostic support system should try to avoid.
- Nambu et al. [61]: in this work, a data augmentation preprocessing is performed to balance the classes. However, for some classes, the number of samples is multiplied by almost 20 (i.e., 19 augmented images are obtained for each original image); therefore, as the augmented images share features on multiple occasions, this could facilitate the convergence task of the classifier.

The works that use a binary classifier are those developed by: Xu et al. [39], Jeftic et al. [42], Sanyal et al. [43], Teramoto et al. [45], Sornapudi et al. [46], Alyafeai et al. [41], one of the classifiers of Isidoro et al. [51], Sanyal et al. [44], Bao et al. [47], Elakkiya et al. [40], one of the classifiers of Manna et al. [52], one of the classifiers of Zhu et al. [53], Kanavati et al. [50], Mulmule et al. [48], and Nagadeepa et al. [49].

Among all of them, the works developed by Jeftic et al. [42], Isidoro et al. [51], Zhu et al. [53], Kanavati et al. [50], Mulmule et al. [48], Nagadeepa et al. [49] do not use a CNN model.

And the works with results most similar to our classifier are those developed by Elakkiya et al. [40], Manna et al. [52]. However, these works have a very important difference: None of them use liquid-based cytology images. For this reason, the classifier complexity may clearly vary. In fact, both classifiers are based on computationally more expensive neural networks than the classifier used in this paper. Therefore, in addition to these two works, others with high classification results using liquid-based cytology are included (works with accuracy greater than 90%). To highlight these differences, we summarize the type of CNN and the number of layers of each type used in Table 10.

In Table 10, we can see that the two studies that do not use liquid cytology samples require models with a much higher computational cost than the other studies. Compared to the others, our work has the lowest number of dense layers and maxpolling layers, but not the lowest number of convolutions. This last parameter is surpassed by the works developed by Sanyal et al. [43, 44], which use 4 and 5 convolutional layers, respectively. However, these two works present deficiencies: Sanyal et al. [43] does not have a dropout layer and, observing the accuracy evolution, an overfitting phenomenon may be occurring; and, Sanyal et al. [44] uses an unbalanced dataset, which may affect the results. Even if we do not take these facts into account, the classifier developed in this work improves the results of both articles by 5%.

On the other hand, the works that implement a classifier with more than 2 classes are Zhang et al. [55], one of the classifiers of Isidoro et al. [51], Zhang et al. [56], Hussain et al. [57], Kuko et al. [60], Martínez-Más et al. [58], one of the classifiers of Manna et al. [52], one of the classifiers of Zhu et al. [53], two classifiers of Kundu et al. [59], Nambu et al. [61], Huang et al. [54].

Among all of them, the classifiers developed by Isidoro et al. [51] and Zhu et al. [53], do not use a CNN model and are not included in the comparison. So, observing the results obtained from the other works, it can be seen that two of the previous articles obtained better accuracy results than this work. This is the case for Manna et al. [52], Kundu et al. [59]. However, the difference does not exceed 2% accuracy.

**Table 11:** Computational complexity for classifiers of 3 or more classes, regarding the number of layers used in the CNN

Work	Model	N_CL_	N_ML_	N_DL_
Hussain et al. [57]	GoogleNet	60	13	6
Kuko et al. [60]	Custom CNN			
Martínez-Más et al. [58]	AlexNet	5	3	3
Manna et al. [52]	Ensemble (Inception-v3 + Xception + DenseNet-201)	200+	20+	10+
Kundu et al. [59]	Ensemble (Inception-v3 + DenseNet-161)	100+	10+	10+
Nambu et al. [61]	ResNeSt	45	5	3
This work	Custom CNN	8	4	3

In the case of works that use more than one model, the less-complex one is indicated; but, in the case of works that use ensembles, the summation of the layers is shown

First, it is important to note that the works developed by Zhang et al. [55, 56], Manna et al. [52] do not use liquid cytology imaging and, for two of them, the accuracy results are very low. So, only the third paper will be included in the comparison.

Regarding the classes used, the classifiers of Hussain et al. [57], Kundu et al. [59] use the same dataset and the same classes as the classifier developed in this work. On the other hand, the classifiers in Kuko et al. [60], Martínez-Más et al. [58] use the same nomenclature (from the Bethesda system), but do not include the carcinoma category. Finally, the classifier developed in the work Nambu et al. [61] is the only one that classifies the five classes of the Bethesda System.

Other shortcomings detected in these studies are detailed below.

- Classifiers presented on Martínez-Más et al. [58] and Kundu et al. [59] train with unbalanced datasets.
- Classifier from Nambu et al. [61] performs a data augmentation process that is too extensive (detailed previously).
- Work developed in Kuko et al. [60] has problematic cases of false positives of the “normal” class.
- The work developed in Manna et al. [52] presents the results obtained for the training set (not the test one).

Finally, to compare the computational cost, the type of CNN and the number of layers of each type used is summarized in Table 11.

As can be observed in Table 11, the two works with the least computational cost are Kuko et al. [60] and Martínez-Más et al. [58]; however, both works present two main issues: first, its accuracy value is low compared to most of the other works; and second, they do not include the carcinoma class in the detection.

For the other works, regardless of their shortcomings, all of them have a much higher computational complexity than the classifier implemented in this work (in the order of 5 times higher). This difference becomes even greater if we compare our classifier with the two classifiers that obtain a slightly better accuracy value than ours: Manna et al. [52] and Kundu et al. [59]. Both use the same classes as this work but, in both cases, to obtain these high accuracy values (exceeding 1.73% and 2% this work, respectively), they require a much higher number of convolutional layers than those used in our work (25 and 12 times more, respectively). Even so, the work Manna et al. [52] presents the results obtained with the training subset.

Thus, after a detailed and extensive discussion, it can be concluded that the only work whose classification results improve this work is the one developed by Kundu et al. [59] (but only by 2%). And analyzing it at the level of computational requirements, it requires more than 12 times more resources than those required by our work.

Therefore, if not only the final accuracy result is taken into account, but also the efficiency ratio with respect to computational resources, our work shows the best results.

### Report generation

The above results show that the system developed in this work obtains an excellent classification result and requires less computational load than in previous works. However, for the report provided to the pathologist, this work gives additional information about the confidence (in percentage value) of the results provided and a heat map that specifies the areas of the image that have been taken into account for the classification. It is based on the application of explainable artificial intelligence (xAI) techniques to extract information from the intermediate layers of the convolutional neural network.

This final report provided to the healthcare professional is of utmost importance, as it will be thoroughly checked to assess the reliability of the results and to consider whether a reevaluation of the sample is necessary.

**Fig. 8:**
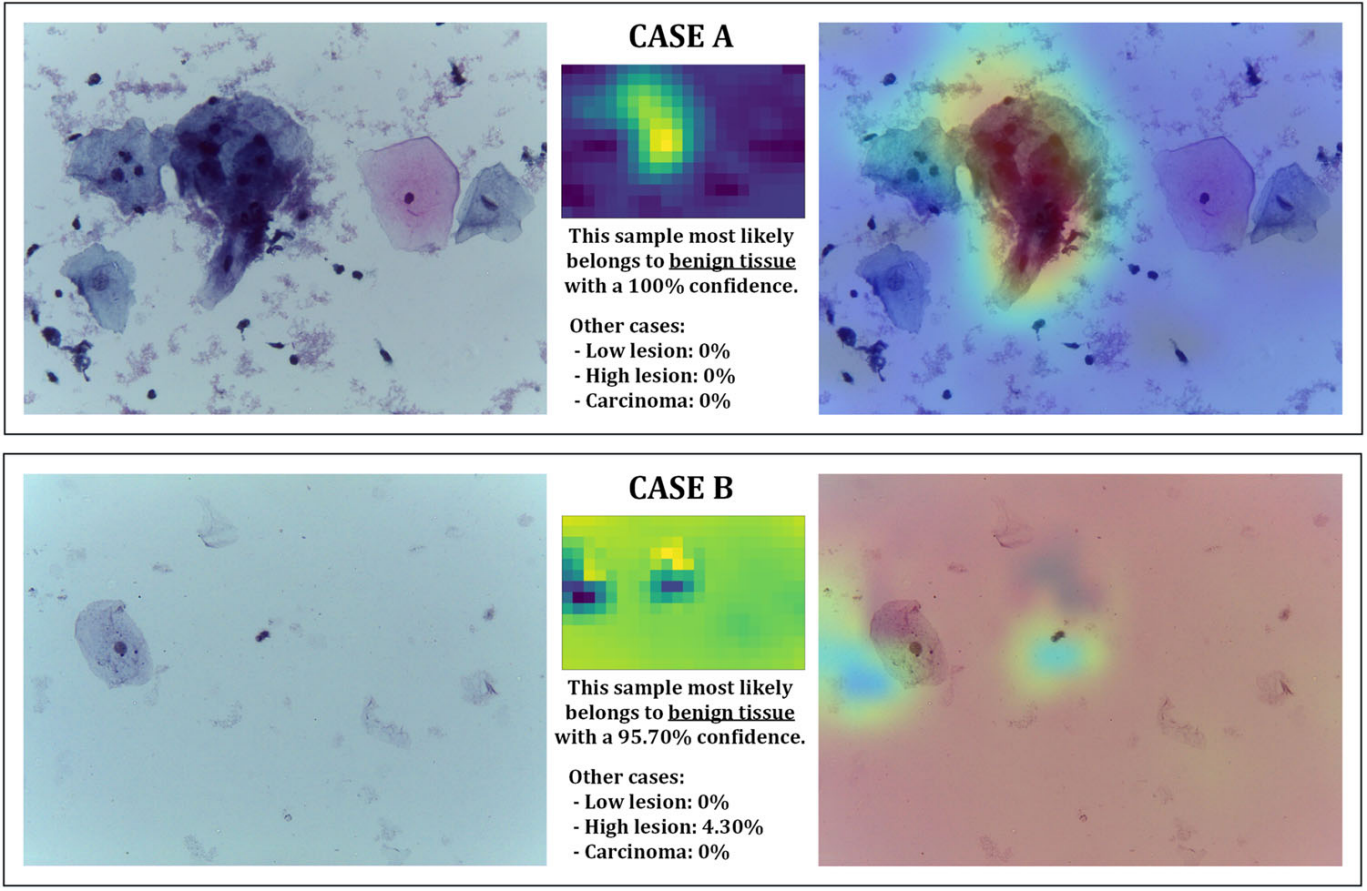
Reports obtained from NEG class samples: (top) one of the best cases with a 100% confidence; (bottom) the worst case with a 95.7% confidence

In this work, the implemented explainable deep learning algorithm (custom Grad-CAM) extracts the resulting information after the last convolution (numerical weight matrix) and converts it to a heat map. This map shows the areas where the classifier has focused to obtain the diagnostic proposal. In the final report, this heat map is overlapped with the original image so that the health professional can appreciate the areas that determine the verdict (see Fig. 4). In addition, the numerical result of the classifier is extracted from the last layer of the system before applying the softmax process (activation of the class with the highest value and inhibition of the remaining ones); in this way, a percentage of reliability of the result can be provided. Based on these parameters, the healthcare professional can make the final verdict, which could be to validate these results or to proceed with a more detailed study of the sample.

It is important to note that the input image has a resolution of $240 \times 180$ pixels, while the heat map has a resolution of $22 \times 14$ pixels (the result of the last convolution layer before maxpooling, see “conv8” layer of the first classifier of Fig. 3). Because of this, the heat map image must be overscaled before overlapping it with the original. This causes that, due to the decimals obtained during this process of resolution increase, some parts of the heat map do not fit perfectly with the original; however, when observing them, it is clear which parts of the image it refers to.

The results of the training dataset will be shown below. Several cases will be shown for each class, with special emphasis on cases with low percentage reliability and classifier system confusion.

#### Negative cases reports

The accuracy obtained by the classifier for this class is perfect (100%), this means that all the cases are classified correctly. The vast majority of classifications give confidence percentages higher than 99%, and there are three cases with lower confidence (98.84, 97.49, and 95.70%).

In Fig. 8, the reports obtained for two samples are shown. The case presented in Fig. 8(top) represents one of the cases with higher confidence (there is more than one sample classified with 100% confidence); and the case presented in Fig. 8 (bottom) represents the case with lower confidence (worst case). In the cases with a confidence 100% in belonging to the class, the confidence regarding the other three classes is zero; however, for the other cases, the report gives a low percentage value for the other classes.

**Fig. 9:**
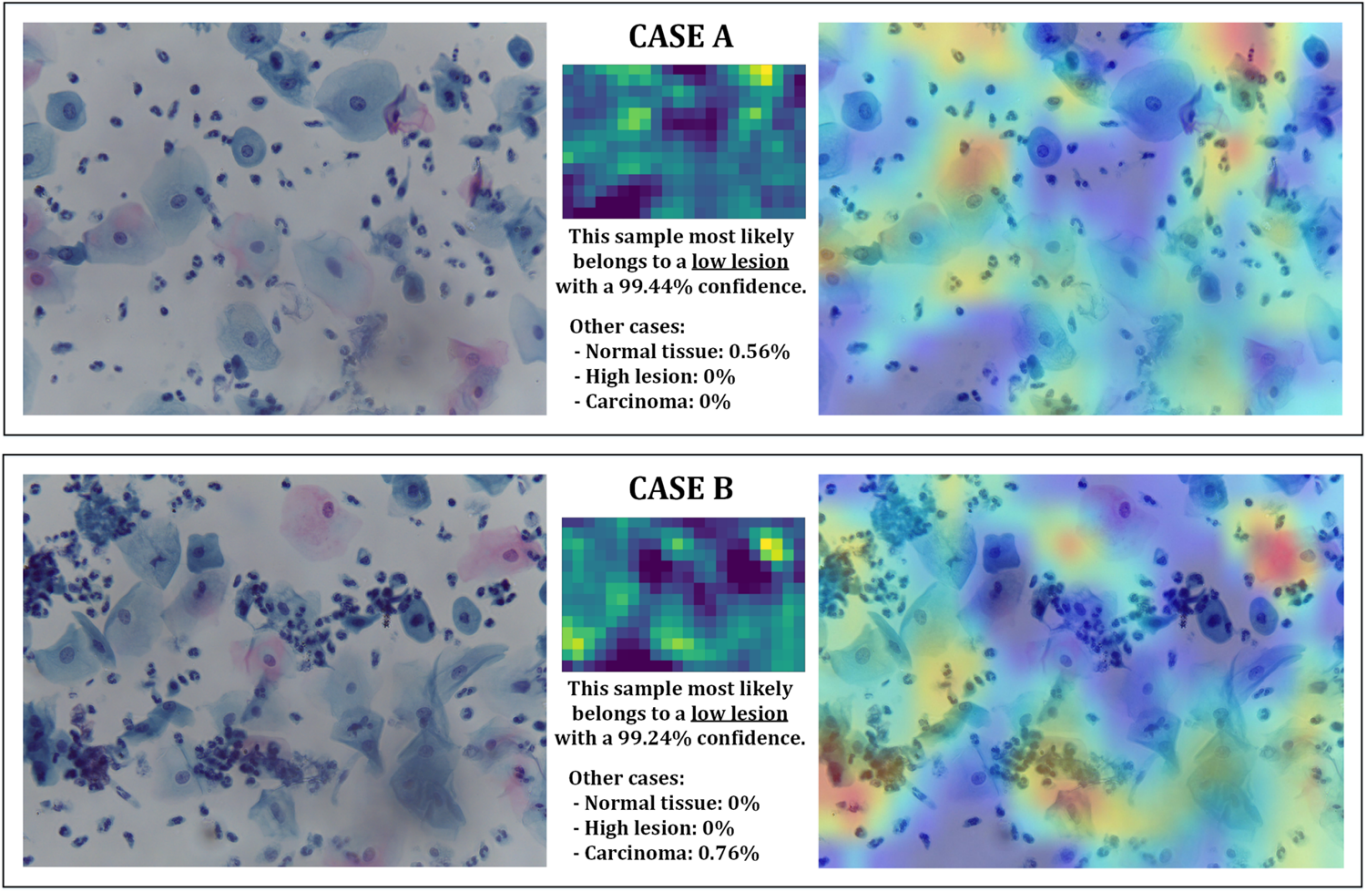
Reports obtained from LOW-class samples: both cases but differentiated due to lymphocytes concentration

If we try to understand the classifier’s criteria for selecting one area of the image or another, we can observe two clearly differentiated cases among all the heat maps of the complete test subset (of which the samples shown in Fig. 8 are its main exponents):

- Samples with solid, large cell concentrations and showy shades: This is the case shown in Fig. 8(top), where the classifier concentrates mainly on areas with cells. In these cases, the accuracy results are higher than 99. 5% for all samples in the test set.
- Samples with low or no cell concentration: this is the case shown in Fig. 8(bottom), where the classifier, having no cells to focus on, focuses its attention on the background of the sample, looking for uniformity. This phenomenon only occurs in the samples of this class and in some sections of the samples of the HIGH class (that is why, in the Fig. 8(bottom) report, a 4.30% confidence of belonging to the HIGH class is indicated).

For this class and the test set used, there are no circumstances in which the confidence percentage is too low; but if it were the case, a low percentage could be a warning sign for the health professional to manually analyze such a case.

#### Low lesion cases reports

The accuracy obtained for this class is also 100%; this means that all samples are correctly classified and, furthermore, no sample from another class is classified as this one. In the reports for all samples, no cases with a confidence result of 100% are found; however, the results obtained for all samples range between 99.24 and 99.99%, making it a more stable class than the previous one. The reports of two samples that represent two different cases are presented in Fig. 9.

In this figure, it can be seen that the samples of this class have the particularity of still presenting large cell forms (similar to the samples of the NEG class) but include a higher concentration of lymphocytes (blue particles), which are immune cells. Based on how this concentration is found in the sample, there are two cases (depicted in Fig. 9):

- Samples with a dispersed lymphocyte concentration: This corresponds to the case in Fig. 9(top). In these situations, with low lymphocyte concentrations, the sample could be mistaken for one of the NEG class. In our case, the classifier distinguishes them all perfectly, but there is a very low percentage of belonging to the NEG class (less than 1%).
- Samples with a concentration of lymphocytes focused on certain areas: this corresponds to the case in Fig. 9 (bottom). In these circumstances, it may be mistaken for a more severe case of the lesion if the classifier looks only at areas with a high concentration of lymphocytes. However, our classifier focuses on the overall concentration (as shown in the heat map) and, therefore, correctly classifies these types of samples as well. However, it can be seen that there is a slight percentage of confidence assigned to the carcinoma class (less than 1%); however, in other samples, this small percentage is distributed between the HIGH and carcinoma classes.

Therefore, in this class, there are no circumstances in the complete set that would lead to confusion of the classifier.

**Fig. 10:**
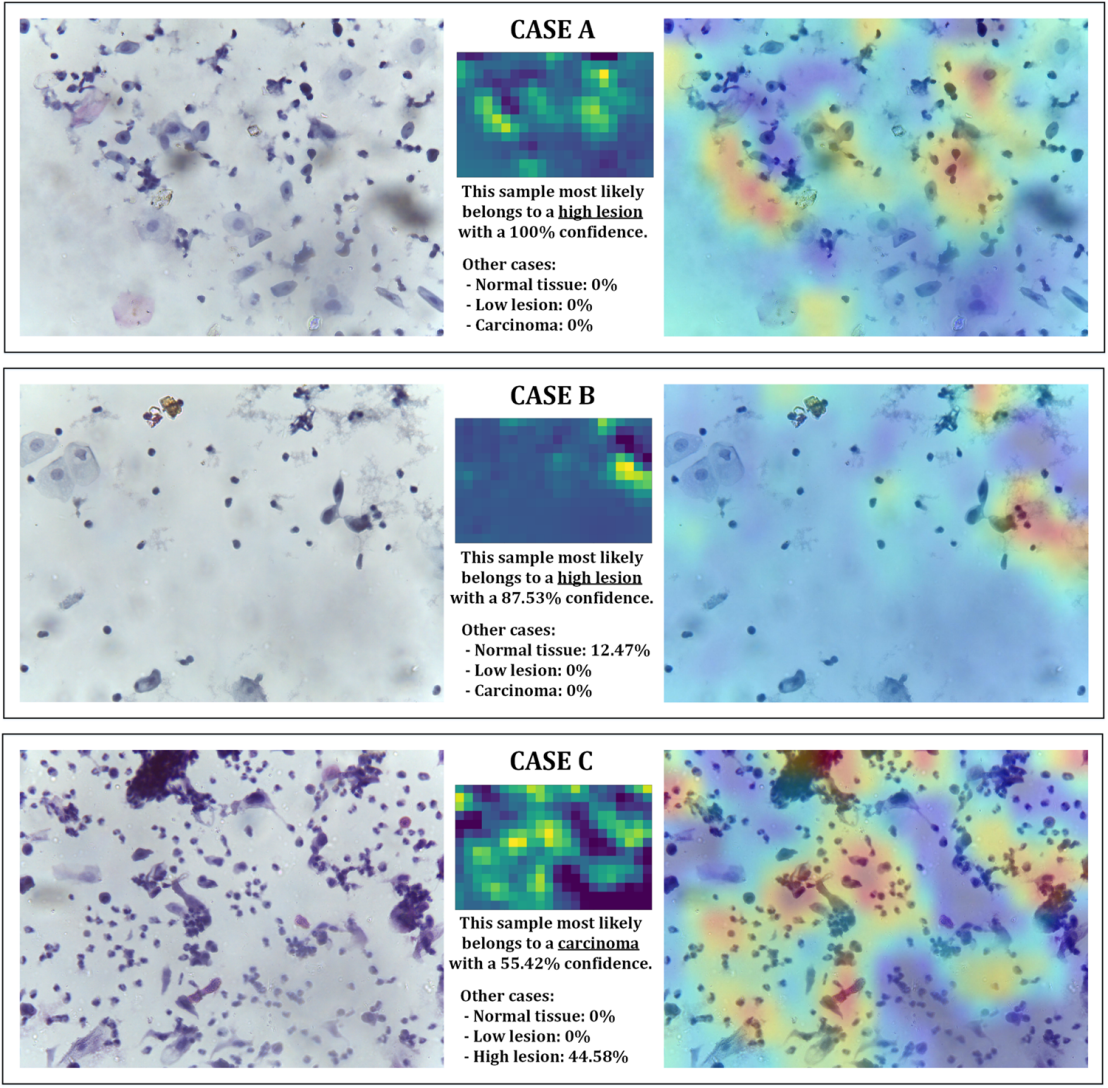
Reports obtained from HIGH-class samples: in this case, three cases are shown depending on the classifier behavior

#### High lesion cases reports

This class is the only one in the dataset with false negatives so that 10% of its samples (3 of 30 in the set used) are incorrectly classified as belonging to the carcinoma class. Thus, the accuracy value of this class is 97.5% (because, although it has false negatives, there are no false positives).

Therefore, in the reports generated for this class, we can find cases of misclassification and, in certain circumstances, correct classifications with a lower percentage of confidence than usual. Figure 10 shows the three different cases that can be found in the entire test suite. These cases are described as follows:

- Samples with a high concentration of lymphocytes, no presence of high cells, and some zones with a blank background: this is the case in Fig. 10(top). These cases are distinguished from NEG class samples due to the large cells that appear in NEG class; and they are distinguished from LOW-class samples due to the fact that the concentration of lymphocytes is more chaotic in HIGH class, including the presence of dispersed blank areas.
- Samples with a medium concentration of lymphocytes and large blank areas: this is the case in Fig. 10(middle). In this case, since the lymphocyte concentration is not uniform, these samples are not mistaken with LOW-class samples; but due to the cases of large blank areas present in some samples of the NEG class, the confidence of these samples is significantly reduced to confidence values from 80 to 90%. However, these samples are correctly classified as they have no large cells (like the samples of the NEG class), but it can be observed in Fig. 10(middle) how the confidence of belonging to the class is reduced to 87.5% (and the rest of the confidence is deposited in the NEG class).
- Samples with a high concentration of lymphocytes without large blank areas: this is the case of Fig. 10(bottom), and these are the samples that are incorrectly classified as belonging to the carcinoma class. As will be seen in the next subsection, this sample resembles the carcinoma class samples. However, the carcinoma class has other elements that distinguish it, but at low concentrations, and that is why, although these samples are classified as belonging to the carcinoma class, the confidence percentage is low. This phenomenon can be seen in Fig. 10 (bottom), where the confidence of belonging to the carcinoma class is 55.42%, while the rest of the confidence is entirely in the HIGH class.

**Fig. 11:**
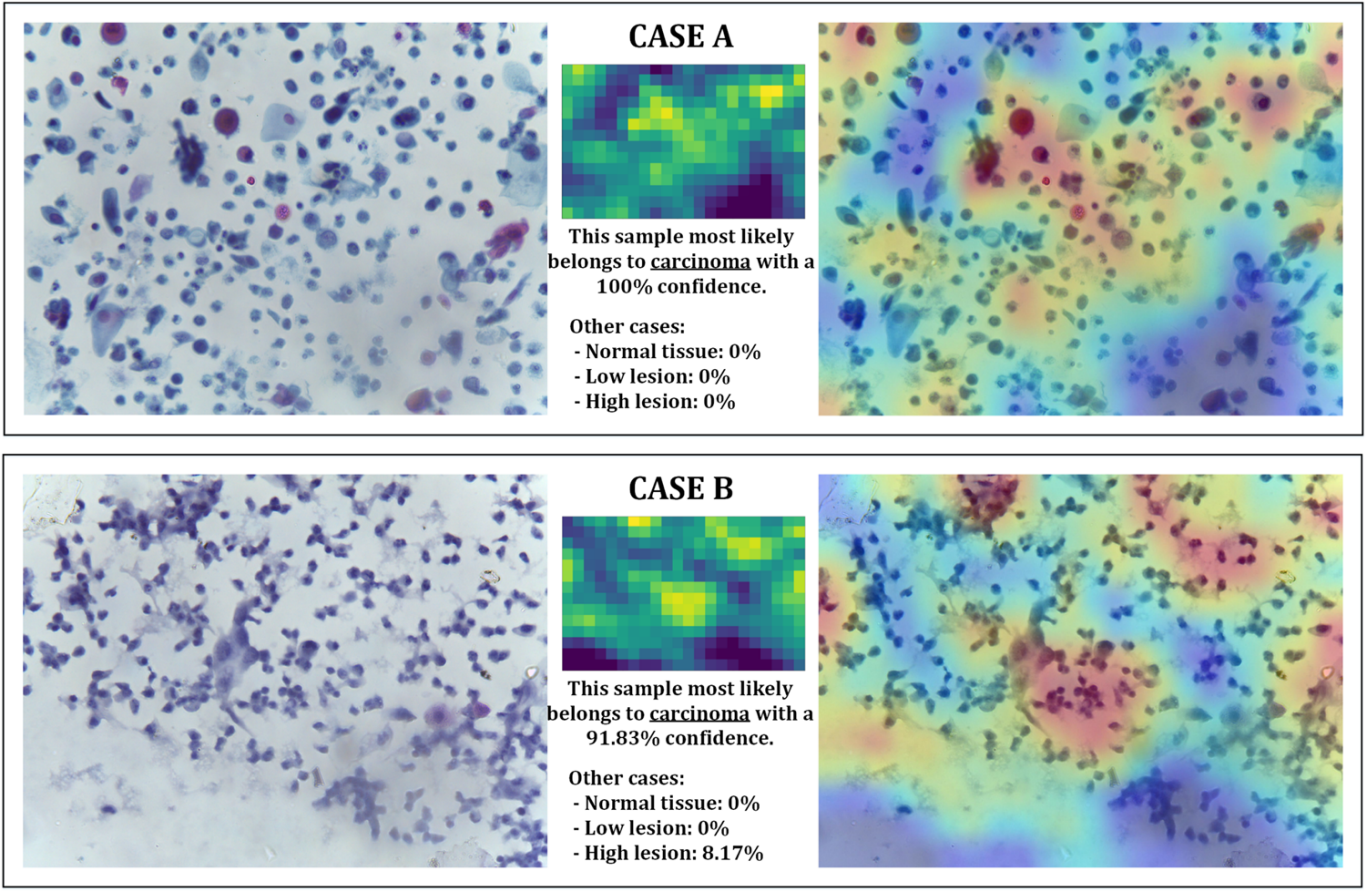
Reports obtained from SCC class samples: two cases with different behaviors

Therefore, the first and only classification faults of the proposed classifier are found in this class. The reason for these failures is due to the similarity of some samples of severe tissue lesions with the presence of carcinoma.

#### Squamous cell carcinoma cases reports

In the latter class, all samples are correctly classified. However, due to false positives caused by samples from the HIGH class (classified as belonging to this class), the accuracy value is reduced to 97.5%.

Therefore, the reports of all the samples in the test set obtain confidence percentages between 91.83 and 100%. Both extreme cases are shown in Fig. 11. On this occasion, we find that all the samples contained in the testing subset are divided into two distinct cases:

- Samples with a high concentration of lymphocytes, no blank areas, and the presence of some red cells: this is the case in Fig. 11(top). In these samples, the classifier perfectly distinguishes the class to which they belong. It is true that in certain samples of the HIGH class there was also a high concentration of lymphocytes, but usually, large bank areas could also be observed; it was only in cases where these areas were not distinguishable that the classifier was wrong (and assumed that they were carcinoma samples). However, in this case, the presence of red cells is decisive for the system to give 100% confidence in the classification.
- Samples with a high concentration of lymphocytes and some large blank areas: this is the case in Fig. 11(bottom). These samples show characteristics similar to those of some samples in the HIGH class (such as the appearance of blank areas), but if the sample in Fig. 10(bottom) is compared with the sample in Fig. 11(bottom), it can be seen that the concentration of lymphocytes in the SCC class is higher, with areas of very high concentration overlapping and blurring. Not surprisingly, although these samples are correctly classified, they are the ones that obtain the lowest percentage of confidence in the final report (values between 91.83 and 95%), with the rest of the confidence given to the HIGH class.

Ultimately, this class is classified correctly and only presents problems of false positives due to the similarities of some samples from the HIGH class.

At this point, the analysis of each class individually has been completed; however, it is interesting to recapitulate and aggregate the observed behavior to detail the overall classification criteria according to the samples and reports analyzed.

In general, samples with a large cell presence and a uniform background are classified as normal. For the other classes, it is the concentration of lymphocytes that distinguishes them; however, in low lesions, large cells are also found on certain occasions (which helps to distinguish these samples from high lesions). In cases of high concentration, this is where the classifier errors are caused by assuming that severe cases of the HIGH class belong to the carcinoma class.

This complete analysis is possible thanks to the application of explainable deep learning techniques on the developed classifier, and it is essential information for the healthcare professional who, in cases of low system confidence, may decide to carry out a personal study to verify or refute the diagnosis made by the classifier. Therefore, this shows that this type of technique brings added value to diagnostic support systems and can help drastically reduce the time required for mass screening of the population.

## Conclusions

In this work, a detailed study regarding the detection of cervical cancer using liquid cytology images and analyzed with artificial intelligence techniques has been performed. A bibliographic study has been carried out to obtain information on advances in the area and to use this study as a starting point for this work.

Based on it, two convolutional neural network-based classifiers (for 2 and 4 classes) have been developed to facilitate mass screening of the population using images obtained from liquid cytology samples, providing a classification based on the Bethesda system.

The results obtained have been compared with the previous works, studying both the efficiency of the classifiers and their complexity. It can be seen that the 2-class classifier obtains an accuracy result of 100% with an AUC of 100%, which improves all the 2-class classifiers of previous works. And the developed 4-class classifier obtains an accuracy result close to 98% with AUC values from 97.4 to 100% depending on the class. These results improve those obtained in previous works with one single exception; but, for that case, our work is more efficient (as it uses less than 10% computational resources).

Finally, this work applies xAI techniques to present a detailed report to the health professionals to help in the diagnosis task: it includes a graphical representation of the heat map showing the areas of the image that have triggered the classification result and a detailed report of the confidence of the system in the results provided. This report generator module is an essential addition to this type of system, as it provides very useful information for the healthcare professional. Thanks to it, the pathologist will be able to ensure the veracity of the results, and, reducing the time needed to perform mass screening in the population.

In summary, the four strengths of this work are the systematic optimization process, the high classification results, the low computational requirements required, and the detailed report generation.

Regarding the limitations of the proposed classifiers, it should be noted that the dataset used does not have a large number of images, it is partially unbalanced and all the images were taken from the same medical instruments. Therefore, data augmentation techniques had to be applied to solve these problems. Even so, in order to consolidate the results obtained, it would be essential to carry out this study on a larger dataset, that include images from different hospitals, in order to avoid the use of data augmentation techniques.

Finally, thanks to the good results obtained in the study of execution times carried out, a new research branch is opened as a continuation of this work, centered on the integration of the classifier developed in an embedded system.

### Supplementary Information

Below is the link to the electronic supplementary material.

Supplementary file 1 (pdf 647 KB)

## References

[1] Alba A et al (2009) The human papillomavirus (HPV) in human pathology: description, pathogenesis, oncogenic role, epidemiology and detection techniques. Open Dermatol J 3(1)

[2] Bruni L et al (2021) Human papillomavirus and related diseases in Africa. Summary report. Technical report, ICO/IARC Information Centre on HPV and Cancer (HPV Information Centre). https://hpvcentre.net/statistics/reports/XFX.pdf

[3] Sung H et al (2021) Global cancer statistics 2020: GLOBOCAN estimates of incidence and mortality worldwide for 36 cancers in 185 countries. CA Cancer J Clin 71(3):209–249 33538338 10.3322/caac.21660

[4] Rohr U-P et al (2016) The value of in vitro diagnostic testing in medical practice: a status report. PloS ONE 11(3):0149856 10.1371/journal.pone.0149856 PMC4778800 26942417

[5] Wilson M et al (2018) Access to pathology and laboratory medicine services: a crucial gap. Lancet 391(10133):1927–1938 29550029 10.1016/S0140-6736(18)30458-6

[6] Madabhushi A, Lee G (2016) Image analysis and machine learning in digital pathology: challenges and opportunities. Med Image Anal 33:170–175 27423409 10.1016/j.media.2016.06.037 PMC5556681

[7] Faust O et al (2018) Deep learning for healthcare applications based on physiological signals: a review. Comput Methods Programs Biomed 161:1–13 29852952 10.1016/j.cmpb.2018.04.005

[8] Torres-Soto J et al (2020) Multi-task deep learning for cardiac rhythm detection in wearable devices. NPJ Digit Med 3(1):1–8 32964139 10.1038/s41746-020-00320-4 PMC7481177

[9] Rim B et al (2020) Deep learning in physiological signal data: a survey. Sensors 20(4):969 32054042 10.3390/s20040969 PMC7071412

[10] Zhu H et al (2020) A deep learning approach for recognizing activity of daily living (ADL) for senior care: exploiting interaction dependency and temporal patterns. Forthcoming at MIS Q

[11] Wang J et al (2019) Deep learning for sensor-based activity recognition: a survey. Pattern Recognition Letters 119:3–11

[12] Nweke HF et al (2018) Deep learning algorithms for human activity recognition using mobile and wearable sensor networks: state of the art and research challenges. Expert Syst Appl 105:233-261

[13] Escobar-Linero E, Domínguez-Morales M, Sevillano JL (2022) Worker’s physical fatigue classification using neural networks. Expert Syst Appl 198:116784

[14] Liu Z et al (2021) Deep learning framework based on integration of S-Mask R-CNN and Inception-v3 for ultrasound image-aided diagnosis of prostate cancer. Future Gener Comput Syst 114:358–367

[15] Syrykh C et al (2020) Accurate diagnosis of lymphoma on whole-slide histopathology images using deep learning. NPJ Digit Med 3(1):1–8 32377574 10.1038/s41746-020-0272-0 PMC7195401

[16] Roncato C et al (2020) Colour doppler ultrasound of temporal arteries for the diagnosis of giant cell arteritis: a multicentre deep learning study. Clin Exp Rheumatol 38(Suppl 124):120–25 32441644

[17] Kundu R et al (2021) Pneumonia detection in chest x-ray images using an ensemble of deep learning models. PloS ONE 16(9):0256630 10.1371/journal.pone.0256630 PMC8423280 34492046

[18] Lotter W et al (2021) Robust breast cancer detection in mammography and digital breast tomosynthesis using an annotation-efficient deep learning approach. Nat Med 27(2):244–249 33432172 10.1038/s41591-020-01174-9 PMC9426656

[19] Thomas SM et al (2021) Interpretable deep learning systems for multi-class segmentation and classification of non-melanoma skin cancer. Med Image Anal 68:101915 33260112 10.1016/j.media.2020.101915

[20] Wei T, Li X, Stojanovic V (2021) Input-to-state stability of impulsive reaction-diffusion neural networks with infinite distributed delays. Nonlinear Dyn 103(2):1733–1755

[21] Cheng P, He S, Stojanovic V, Luan X, Liu F (2021) Fuzzy fault detection for Markov jump systems with partly accessible hidden information: an event-triggered approach. IEEE Trans Cybern 10.1109/TCYB.2021.3050209 33513123

[22] Civit-Masot J et al (2020) Deep learning system for COVID-19 diagnosis aid using x-ray pulmonary images. Appl Sci 10(13):4640

[23] Luna-Perejón F et al (2019) Wearable fall detector using recurrent neural networks. Sensors 19(22):4885 31717442 10.3390/s19224885 PMC6891713

[24] Muñoz-Saavedra L, Escobar-Linero E, Civit-Masot J, Luna-Perejón F, Civit A, Domínguez-Morales M (2023) A robust ensemble of convolutional neural networks for the detection of monkeypox disease from skin images. Sensors 23(16):7134 37631672 10.3390/s23167134 PMC10459252

[25] Wright A et al (2018) Clinical decision support alert malfunctions: analysis and empirically derived taxonomy. J Am Med Inform Assoc 25(5):496–506 29045651 10.1093/jamia/ocx106 PMC6019061

[26] Von-Eschenbach WJ (2021) Transparency and the black box problem: why we do not trust AI. Philos Technol 1–16

[27] Singh A et al (2020) Explainable deep learning models in medical image analysis. J Imaging 6(6):52 34460598 10.3390/jimaging6060052 PMC8321083

[28] Angelov P, Soares E (2020) Towards explainable deep neural networks (xDNN). Neural Netw 130:185–194 32682084 10.1016/j.neunet.2020.07.010

[29] Xue Q, Chuah MC (2019) Explainable deep learning based medical diagnostic system. Smart Health 13:100068

[30] Brunese L et al (2020) Explainable deep learning for pulmonary disease and coronavirus COVID-19 detection from x-rays. Comput Methods Programs Biomed 196:105608 32599338 10.1016/j.cmpb.2020.105608 PMC7831868

[31] Muñoz-Saavedra L, Escobar-Linero E, Miró-Amarante L, Bohórquez MR, Domínguez-Morales M (2023) Designing and evaluating a wearable device for affective state level classification using machine learning techniques. Expert Syst Appl 219:119577

[32] Luna-Perejón F et al (2020) Low-power embedded system for gait classification using neural networks. J Low Power Electron Appl 10(2):14

[33] Escobar-Linero E, Luna-Perejón F, Muñoz-Saavedra L, Sevillano JL, Domínguez-Morales M (2022) On the feature extraction process in machine learning. An experimental study about guided versus non-guided process in falling detection systems. Eng Appl Artif Intell 114:105170

[34] Civit-Masot J, Bañuls-Beaterio A, Domínguez-Morales M, Rivas-Pérez M, Muñoz-Saavedra L, Corral JMR (2022) Non-small cell lung cancer diagnosis aid with histopathological images using explainable deep learning techniques. Comput Methods Programs Biomed 226:107108 36113183 10.1016/j.cmpb.2022.107108

[35] Hussain E et al (2020) Liquid based-cytology pap smear dataset for automated multi-class diagnosis of pre-cancerous and cervical cancer lesions. Data Brief 30:105589 32368601 10.1016/j.dib.2020.105589 PMC7186519

[36] Sokolova M, Lapalme G (2009) A systematic analysis of performance measures for classification tasks. Inf Process Manag 45(4):427–437

[37] Hoo ZH, Candlish J, Teare D (2017) What is an ROC curve? Emerg Med J 34(6):357–359 28302644 10.1136/emermed-2017-206735

[38] WHO (2006) Comprehensive cervical cancer control: a guide to essential practice. World Health Organization, Switzerland 25642554

[39] Xu T et al (2017) Multi-feature based benchmark for cervical dysplasia classification evaluation. Pattern Recognit 63:468–475 28603299 10.1016/j.patcog.2016.09.027 PMC5464748

[40] Elakkiya R et al (2021) Cervical cancer diagnostics healthcare system using hybrid object detection adversarial networks. IEEE J Biomed Health Inform 10.1109/JBHI.2021.3094311 34214045

[41] Alyafeai Z et al (2020) A fully-automated deep learning pipeline for cervical cancer classification. Expert Syst Appl 141:112951

[42] Jeftic B et al (2019) Machine learning classification of cervical tissue liquid based cytology smear images by optomagnetic imaging spectroscopy. Teh Vjesn 26(6):1694–1699

[43] Sanyal P et al (2019) Performance of a convolutional neural network in screening liquid based cervical cytology smears. J Cytol 36(3):146 31359913 10.4103/JOC.JOC_201_18 PMC6592125

[44] Sanyal P et al (2020) Performance characteristics of an artificial intelligence based on convolutional neural network for screening conventional Papanicolaou-stained cervical smears. Med J Armed Forces India 76(4):418–424 33162650 10.1016/j.mjafi.2019.08.001 PMC7606095

[45] Teramoto A et al (2019) Automated classification of benign and malignant cells from lung cytological images using deep convolutional neural network. Inform Med Unlocked 16:100205

[46] Sornapudi S et al (2019) Comparing deep learning models for multi-cell classification in liquid-based cervical cytology image. In: AMIA Annual Symposium Proceedings, vol 2019. American Medical Informatics Association, pp 820 PMC7153123 32308878

[47] Bao H et al (2020) Artificial intelligence-assisted cytology for detection of cervical intraepithelial neoplasia or invasive cancer: a multicenter, clinical-based, observational study. Gynecol Oncol 159(1):171–178 32814641 10.1016/j.ygyno.2020.07.099

[48] Mulmule PV, Kanphade RD, Dhane DM (2022) Artificial intelligence-assisted cervical dysplasia detection using Papanicolaou smear images. Vis Comput 1–12

[49] Nagadeepa C, Sai PP, Madhuri G, Reddy KS, Reddy DVB (2022) Artificial intelligence based cervical cancer risk prediction using m1 algorithms. In: 2022 International conference on emerging smart computing and informatics (ESCI). IEEE, pp 1–6

[50] Kanavati F, Hirose N, Ishii T, Fukuda A, Ichihara S, Tsuneki M (2022) A deep learning model for cervical cancer screening on liquid-based cytology specimens in whole slide images. Cancers 14(5):1159 35267466 10.3390/cancers14051159 PMC8909106

[51] Isidoro DW et al (2020) Automatic classification of cervical cell patches based on non-geometric characteristics. In: VISIGRAPP (5: VISAPP), pp 845–852

[52] Manna A et al (2021) A fuzzy rank-based ensemble of CNN models for classification of cervical cytology. Sci Rep 11(1):1–18 34267261 10.1038/s41598-021-93783-8 PMC8282795

[53] Zhu X et al (2021) Hybrid AI-assistive diagnostic model permits rapid TBS classification of cervical liquid-based thin-layer cell smears. Nat Commun 12(1):1–12 34112790 10.1038/s41467-021-23913-3 PMC8192526

[54] Huang P, Zhang S, Li M, Wang J, Ma C, Wang B, Lv X (2020) Classification of cervical biopsy images based on LASSO and EL-SVM. IEEE Access 8:24219–24228

[55] Zhang X et al (2019) Cervical image classification based on image segmentation preprocessing and a CapsNet network model. Int J Imaging Syst Technol 29(1):19–28

[56] Zhang T et al (2020) Cervical precancerous lesions classification using pre-trained densely connected convolutional networks with colposcopy images. Biomed Signal Process Control 55:101566

[57] Hussain E et al (2020) A comprehensive study on the multi-class cervical cancer diagnostic prediction on pap smear images using a fusion-based decision from ensemble deep convolutional neural network. Tissue Cell 65:101347 32746984 10.1016/j.tice.2020.101347

[58] Martínez-Más J et al (2020) Classifying Papanicolaou cervical smears through a cell merger approach by deep learning technique. Expert Syst Appl 160:113707

[59] Kundu R et al (2021) Ensemble of CNN classifiers using Sugeno fuzzy integral technique for cervical cytology image classification. arXiv:2108.09460

[60] Kuko M et al (2020) Single and clustered cervical cell classification with ensemble and deep learning methods. Inf Syst Front 22(5):1039–1051

[61] Nambu Y et al (2021) A screening assistance system for cervical cytology of squamous cell atypia based on a two-step combined CNN algorithm with label smoothing. Cancer Med 10.1002/cam4.4460 PMC8729059 34841722

[62] Hosmer D Jr et al (2013) Applied logistic regression, vol 398. John Wiley & Sons, New Jersey

[63] Metz C (1978) Basic principles of roc analysis. In: Seminars in nuclear medicine, vol 8(4). Elsevier, pp 283–298 10.1016/s0001-2998(78)80014-2 112681

