# Supplementary material for: A lightweight xAI approach to cervical cancer classification
Source: Med Biol Eng Comput. 2024 Mar 20;62(8):2281–304. doi: 10.1007/s11517-024-03063-6 (PMC11289177; doi:10.1007/s11517-024-03063-6)
Supplement: Supplementary file 1 — (pdf 647 KB) [file 11517_2024_3063_MOESM1_ESM.pdf]

Supplementary Material

**Batch size**	**Learning Rate**															
	**1e-5**		**5e-5**		**1e-4**		**5e-4**		**1e-3**		**5e-3**		**1e-2**		**5e-2**	
	Acc	Loss	Acc	Loss	Acc	Loss	Acc	Loss	Acc	Loss	Acc	Loss	Acc	Loss	Acc	Loss
**2**	57.49	0.94	80.89	0.51	81.84	0.44	84.16	0.70	72.95	1.21	20.21	1.35	28.29	1.34	21.00	1.33
**4**	58.33	0.92	86.01	0.33	95.15	0.25	81.24	1.54	20.19	1.43	19.59	1.38	25.52	1.36	20.78	1.46
**6**	58.44	0.82	90.43	0.30	82.68	0.56	95.48	0.31	20.19	1.43	19.59	1.38	24.50	1.36	25.50	1.43
**8**	69.09	0.65	93.23	0.21	94.00	0.16	89.43	0.60	81.25	0.54	17.81	1.33	18.75	1.40	26.56	1.43
**10**	57.95	0.95	94.92	0.15	86.13	0.31	96.87	0.13	76.95	0.82	19.00	1.32	19.60	1.36	23.75	1.32
**12**	58.50	0.85	94.82	0.15	96.56	0.12	95.33	0.32	90.74	0.69	16.89	1.33	16.82	1.33	24.00	1.35
**14**	78.86	0.31	89.12	0.20	84.05	0.42	84.19	0.51	81.18	0.81	24.20	1.32	24.20	1.32	22.57	1.46
**16**	79.47	0.49	89.16	0.22	95.17	0.14	88.38	0.66	95.99	0.43	18.27	1.40	18.27	1.40	21.89	1.40
**18**	83.00	0.36	94.40	0.12	93.18	0.15	86.53	0.42	82.89	0.42	17.29	1.37	17.12	1.36	27.26	1.43
**20**	83.64	0.44	88.21	0.27	91.08	0.17	81.70	0.48	89.24	0.68	16.66	1.37	17.17	1.41	25.21	1.36
**22**	78.52	0.58	81.03	0.29	92.85	0.19	85.00	0.64	91.92	0.36	18.22	1.47	17.34	1.40	21.86	1.43
**24**	81.47	0.57	91.37	0.15	98.95	0.03	94.56	0.26	80.46	0.93	18.23	1.44	17.17	1.36	22.31	1.43
**26**	88.97	0.34	94.41	0.11	91.59	0.55	89.77	0.34	92.23	1.03	23.23	1.45	22.79	1.42	22.79	1.42
**28**	82.51	0.81	94.78	0.15	97.56	0.12	89.62	0.44	91.30	0.48	25.28	1.38	26.34	1.40	26.34	1.40
**30**	82.49	0.46	94.57	0.17	89.01	0.19	91.74	0.40	87.88	0.19	18.40	1.34	18.39	1.33	19.75	1.44
**32**	86.40	0.35	93.93	0.18	92.14	0.15	96.89	0.10	88.66	0.32	18.59	1.35	19.36	1.40	21.60	1.34

Table 1 8-convolution architecture. Accuracy values (Acc) in % during phase 1. Results presented are the average of five tests performed for each one.

**Batch size**	**Learning Rate**															
	**1e-5**		**5e-5**		**1e-4**		**5e-4**		**1e-3**		**5e-3**		**1e-2**		**5e-2**	
	Acc	Loss	Acc	Loss	Acc	Loss	Acc	Loss	Acc	Loss	Acc	Loss	Acc	Loss	Acc	Loss
**2**	81.47	0.45	69.34	1.81	70.81	1.94	69.06	2.08	68.71	2.01	20.21	1.43	28.29	1.43	22.53	1.35
**4**	65.34	1.19	69.70	1.51	84.37	0.82	86.66	0.81	68.00	2.19	20.58	1.33	26.30	1.39	19.59	1.40
**6**	85.41	0.38	87.97	0,42	84.73	0.71	88.59	0.79	76.80	1.50	20.78	1.33	24.75	1.41	24.25	1.44
**8**	87.31	0.46	96.56	0.16	92.27	0.33	83.94	0.89	83.12	1.44	18.56	1.41	18.94	1.39	25.78	1.42
**10**	85.00	0.38	80.51	0.52	95.00	0.16	83.64	0.83	74.88	1.34	19.20	1.45	20.00	1.39	26.00	1.34
**12**	87.90	0.31	96.70	0.14	90.86	0.25	86.31	0.43	74.77	1.83	18.42	1.34	17.00	1.46	26.00	1.35
**14**	94.89	0.32	94.33	0.18	89.57	0.48	93.39	0.19	89.65	0.39	25.72	1.38	25.22	1.40	21.92	1.39
**16**	90.23	0.30	86.00	0.85	93.29	0.17	78.84	0.72	84.00	1.59	17.54	1.46	18.27	1.40	21.45	1.45
**18**	94.82	0.32	83.76	0.90	83.82	0.37	90.88	0.53	93.73	0.21	17.29	1.43	17.11	1.44	25.68	1.43
**20**	90.89	0.28	90.25	0.19	93.84	0.20	80.64	0.70	86.86	0.64	17.17	1.39	16.49	1.44	24.70	1.47
**22**	88.63	0.29	84.24	0.46	94.72	0.26	96.79	0.15	80.65	0.95	17.34	1.40	17.51	1.39	21.64	1.39
**24**	95.83	0.21	93.44	0.26	94.42	0.27	92.08	0.48	90.94	0.36	17.00	1.46	17.89	1.39	17.18	1.44
**26**	87.71	0.33	77.93	0.61	96.61	0.19	85.72	0.62	79.66	2.26	22.56	1.38	21.89	1.42	21.67	1.43
**28**	91.30	0.25	89.62	0.30	92.55	0.21	87.55	0.50	83.31	2.00	26.08	1.39	25.56	1.42	26.08	1.39
**30**	90.83	0.25	86.49	0.42	87.88	0.28	97.89	0.10	84.94	2.21	76.13	2.00	18.59	1.44	18.79	1.43
**32**	93.34	0.26	90.65	0.22	94.17	0.24	80.68	0.74	83.62	1.33	18.98	1.41	18.97	1.41	23.63	1.33

Table 2 6-convolution architecture. Accuracy values (Acc) in % during phase 1. Results presented are the average of five tests performed for each one.

Batch size	Learning Rate															
	1e-5		5e-5		1e-4		5e-4		1e-3		5e-3		1e-2		5e-2	
	Acc	Loss	Acc	Loss	Acc	Loss	Acc	Loss	Acc	Loss	Acc	Loss	Acc	Loss	Acc	Loss
**2**	88.00	0.27	69.74	1.90	67.38	1.31	66.93	1.55	73.31	1.48	20.41	1.42	28.29	1.43	21.21	1.43
**4**	86.77	0.41	87.50	0.68	80.00	0.68	70.26	0.99	53.09	1.96	20.58	1.34	26.30	1.39	26.56	1.37
**6**	87.09	0.27	85.00	0.74	91.67	0.28	94.53	0.19	86.09	0.48	20.58	1.34	19.39	1.43	26.25	1.33
**8**	96.89	0.15	87.65	0.40	95.75	0.24	82.62	0.89	73.28	2.09	19.50	1.34	18.38	1.42	25.00	1.44
**10**	88.20	0.23	96.60	0.37	88.35	0.17	82.62	0.82	80.36	0.93	20.60	1.35	20.60	1.35	25.00	1.39
**12**	91.79	0.19	96.87	0.12	84.50	0.82	83.00	0.48	88.00	0.82	17.18	1.44	17.36	1.43	24.75	1.40
**14**	94.18	0.30	92.43	0.21	92.23	0.45	90.52	0.59	86.02	0.77	26.23	1.36	24.20	1.46	19.61	1.41
**16**	90.99	0.26	86.77	0.26	84.92	0.53	92.10	0.55	74.61	1.73	78.46	1.75	18.27	1.40	23.22	1.33
**18**	94.96	0.24	88.32	0.33	95.73	0.28	84.82	0.79	85.64	2.18	18.53	1.33	16.94	1.46	25.41	1.45
**20**	94.91	0.26	94.94	0.14	98.46	0.09	79.20	1.00	83.60	0.46	16.66	1.43	17.51	1.36	23.00	1.40
**22**	93.04	0.18	92.65	0.26	96.88	0.18	80.93	1.00	88.63	1.05	17.69	1.37	17.17	1.41	21.43	1.40
**24**	93.87	0.32	97.44	0.19	87.79	0.34	83.87	0.68	71.74	1.47	17.36	1.43	16.82	1.47	18.42	1.34
**26**	90.42	0.23	94.78	0.22	95.49	0.33	79.24	1.00	80.85	1.11	16.51	1.44	23.01	1.35	21.45	1.45
**28**	93.52	0.33	94.56	0.21	93.48	0.24	95.65	0.22	72.06	1.74	18.09	1.34	25.56	1.42	17.91	1.36
**30**	97.20	0.16	91.04	0.19	81.68	0.68	89.17	0.61	70.46	1.59	19.67	1.40	18.79	1.43	18.98	1.41
**32**	92.75	0.29	95.53	0.19	96.25	0.36	76.76	0.99	74.31	1.47	20.13	1.33	20.13	1.33	19.36	1.39

Table 3 4-convolution architecture. Accuracy values (Acc) in % during phase 1. Results presented are the average of five tests performed for each one.

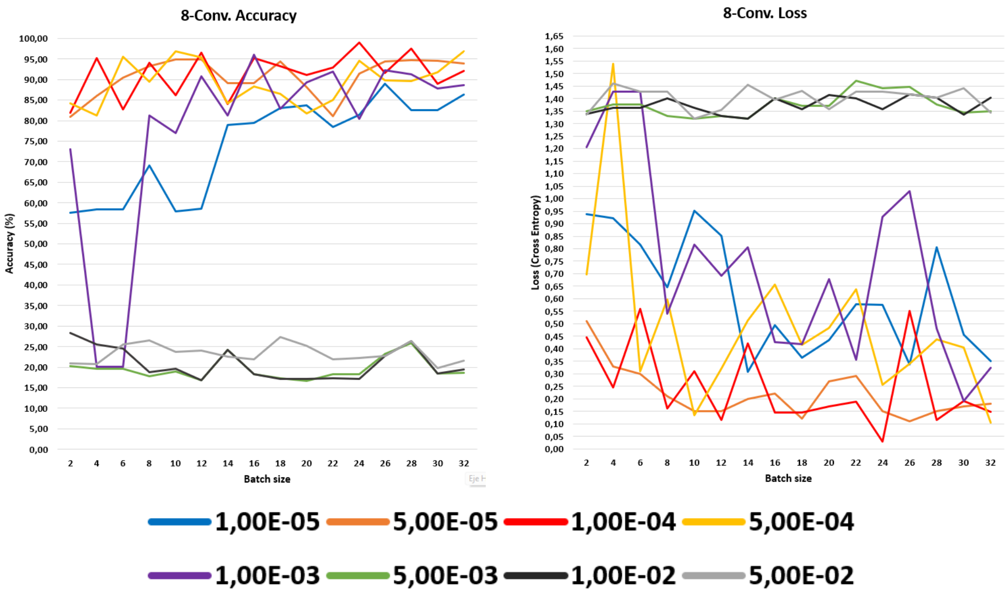

Fig. 1 Accuracy and Loss summary for the 8-convolution architecture during phase 2.

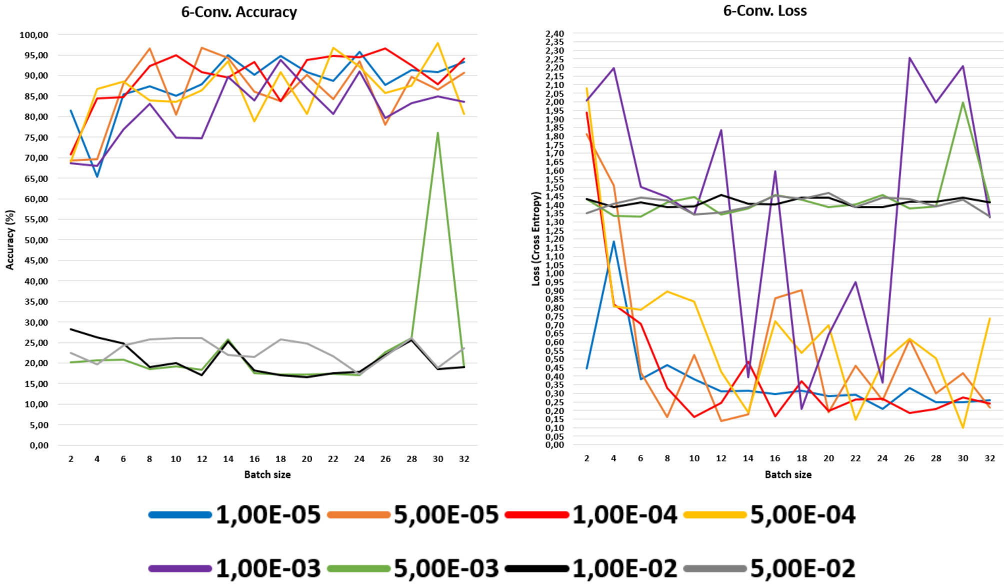

Fig. 2 Accuracy and Loss summary for the 6-convolution architecture during phase 2.

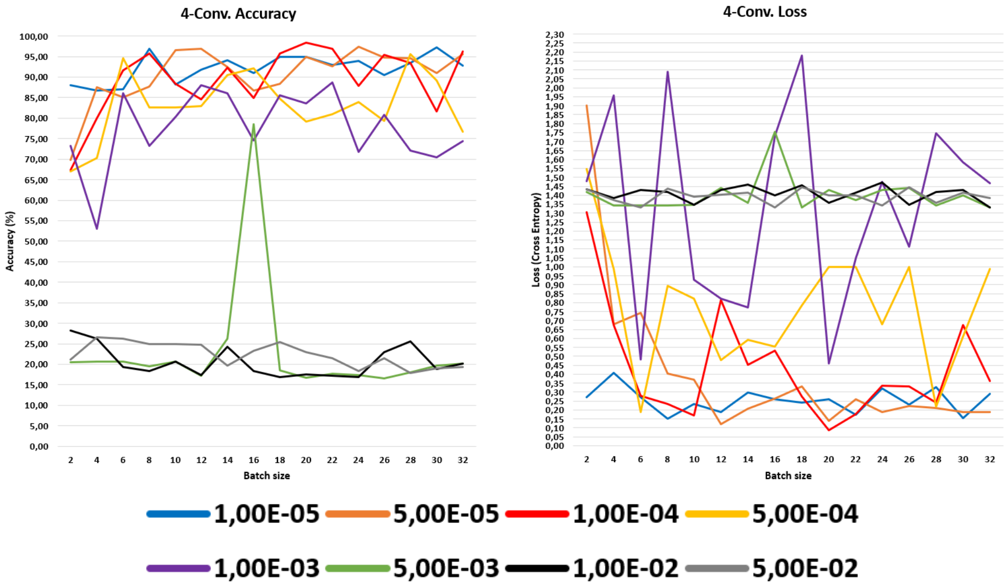

Fig. 3 Accuracy and Loss summary for the 4-convolution architecture during phase 2.

Batch size	*GPU (GeForce GTX 1080Ti)*			*CPU (Intel i7-10700K @ 3.8GHz)*		
	**CNN1**	**CNN2**	**CNN3**	**CNN1**	**CNN2**	**CNN3**
**1**	0.0035±0.0004	0.0028±0.0001	0.0022±0.0000	0.0070±0.0005	0.0062±0.0006	0.0057±0.0002
**2**	0.0033±0.0000	0.0027±0.0000	0.0022±0.0001	0.0102±0.0006	0.0098±0.0006	0.0084±0.0003
**3**	0.0034±0.0003	0.0029±0.0002	0.0022±0.0001	0.0139±0.0004	0.0126±0.0004	0.0116±0.0004
**4**	0.0036±0.0005	0.0028±0.0001	0.0024±0.0002	0.0159±0.0010	0.0142±0.0010	0.0135±0.0008
**5**	0.0038±0.0007	0.0028±0.0001	0.0022±0.0000	0.0221±0.0019	0.0175±0.0010	0.0166±0.0011
**6**	0.0034±0.0002	0.0028±0.0001	0.0022±0.0001	0.0277±0.0022	0.0219±0.0012	0.0208±0.0013
**7**	0.0034±0.0001	0.0030±0.0003	0.0022±0.0001	0.0358±0.0034	0.0274±0.0016	0.0258±0.0015
**8**	0.0034±0.0001	0.0028±0.0001	0.0022±0.0001	0.0342±0.0033	0.0261±0.0016	0.0250±0.0013
**9**	0.0036±0.0006	0.0027±0.0001	0.0022±0.0000	0.0419±0.0017	0.0342±0.0013	0.0327±0.0013
**10**	0.0033±0.0002	0.0028±0.0001	0.0022±0.0001	0.0481±0.0021	0.0392±0.0022	0.0373±0.0015
**11**	0.0034±0.0002	0.0029±0.0002	0.0022±0.0000	0.0565±0.0024	0.0470±0.0022	0.0450±0.0021
**12**	0.0034±0.0002	0.0028±0.0001	0.0022±0.0000	0.0588±0.0028	0.0476±0.0024	0.0451±0.0021
**13**	0.0055±0.0037	0.0032±0.0020	0.0026±0.0018	0.0779±0.0029	0.0594±0.0026	0.0575±0.0023
**14**	0.0059±0.0041	0.0035±0.0027	0.0032±0.0029	0.0894±0.0038	0.0665±0.0035	0.0662±0.0026
**15**	0.0063±0.0042	0.0042±0.0036	0.0037±0.0035	0.0989±0.0036	0.0773±0.0029	0.0774±0.0029
**16**	0.0054±0.0042	0.0033±0.0026	0.0028±0.0022	0.1018±0.0031	0.0731±0.0032	0.0721±0.0026
**17**	0.0061±0.0044	0.0044±0.0034	0.0037±0.0035	0.1226±0.0049	0.0934±0.0032	0.0877±0.0033
**18**	0.0064±0.0043	0.0041±0.0032	0.0034±0.0031	0.1413±0.0085	0.1039±0.0032	0.0960±0.0031
**19**	0.0075±0.0045	0.0045±0.0039	0.0039±0.0038	0.1548±0.0040	0.1154±0.003	0.1070±0.0035
**20**	0.0078±0.0048	0.0049±0.0040	0.0042±0.0040	0.1592±0.0076	0.1193±0.0037	0.1116±0.0035
**21**	0.0094±0.0050	0.0082±0.0054	0.0071±0.0056	0.1727±0.0049	0.1339±0.0068	0.1231±0.0030
**22**	0.0099±0.0052	0.0088±0.0055	0.0081±0.0060	0.1877±0.0051	0.1396±0.0057	0.1387±0.0087
**23**	0.0107±0.0052	0.0094±0.0056	0.0083±0.0059	0.2031±0.0045	0.1512±0.0035	0.1462±0.0035
**24**	0.0112±0.0053	0.0099±0.0057	0.0088±0.0060	0.2083±0.0071	0.1523±0.0036	0.1508±0.0031
**25**	0.0123±0.0053	0.0110±0.0068	0.0098±0.0071	0.2640±0.0064	0.1829±0.0069	0.1743±0.0038
**26**	0.0133±0.0056	0.0119±0.0060	0.0108±0.0064	0.2913±0.0071	0.1980±0.0062	0.1888±0.0032
**27**	0.0139±0.0070	0.0131±0.0060	0.0111±0.0072	0.3102±0.0060	0.2055±0.0038	0.1994±0.0042
**28**	0.0149±0.0072	0.0135±0.0070	0.0118±0.0074	0.3230±0.0071	0.2129±0.0044	0.2041±0.0032
**29**	0.0157±0.0075	0.0134±0.0084	0.0114±0.0075	0.3419±0.0063	0.2310±0.0038	0.2261±0.0057
**30**	0.0169±0.0081	0.0140±0.0084	0.0119±0.0081	0.3596±0.0055	0.2546±0.0060	0.2435±0.0048
**31**	0.0181±0.0088	0.0147±0.0091	0.0124±0.0090	0.3948±0.0094	0.2788±0.0056	0.2618±0.0046
**32**	0.0183±0.0088	0.0165±0.0093	0.0144±0.0093	0.3782±0.0058	0.2729±0.0059	0.2547±0.0046
**33**	0.0261±0.0107	0.0211±0.0117	0.0183±0.0124	0.4682±0.009	0.3241±0.0049	0.3109±0.0092
**34**	0.0271±0.0114	0.0215±0.0124	0.0187±0.0132	0.5095±0.0109	0.3530±0.0059	0.3292±0.0082
**35**	0.0282±0.0121	0.0229±0.0133	0.0207±0.0137	0.5387±0.0119	0.3647±0.0053	0.3448±0.0088
**36**	0.0284±0.0123	0.0234±0.0124	0.0205±0.0133	0.5596±0.0083	0.3907±0.0071	0.3738±0.0115
**37**	0.0307±0.0137	0.0255±0.0145	0.0209±0.0138	0.5912±0.0213	0.4271±0.0056	0.4019±0.0112
**38**	0.0321±0.0138	0.0244±0.0138	0.0202±0.0135	0.6206±0.0196	0.4455±0.0209	0.4167±0.0091
**39**	0.0343±0.0150	0.0248±0.0139	0.0214±0.0146	0.6656±0.0199	0.4704±0.0096	0.4509±0.0112
**40**	0.0320±0.0142	0.0264±0.0152	0.0216±0.0147	0.6411±0.0158	0.4616±0.0195	0.4428±0.0097
**41**	0.0363±0.0154	0.0285±0.0163	0.0261±0.0184	0.7808±0.0178	0.5622±0.0173	0.5417±0.0178
**42**	0.0379±0.0159	0.0292±0.0168	0.0258±0.0181	0.8160±0.0417	0.5600±0.0261	0.5373±0.0105
**43**	0.0402±0.0166	0.0305±0.0175	0.0263±0.0183	0.8252±0.0220	0.5805±0.0117	0.5676±0.0116
**44**	0.0410±0.0170	0.0317±0.0176	0.0276±0.0186	0.8569±0.0272	0.5832±0.0107	0.5886±0.0239
**45**	0.0423±0.0178	0.0330±0.0187	0.0282±0.0194	0.8928±0.0198	0.6322±0.0253	0.6126±0.0197
**46**	0.0444±0.0190	0.0337±0.0189	0.0289±0.0198	0.9444±0.0189	0.6407±0.0117	0.6392±0.0187
**47**	0.0476±0.0204	0.0352±0.0195	0.0301±0.0204	0.9774±0.0210	0.6856±0.0120	0.6702±0.0197
**48**	0.0446±0.0190	0.0343±0.0192	0.0290±0.0198	0.9276±0.0248	0.6515±0.0142	0.6533±0.0114
**49**	0.0516±0.0211	0.0426±0.0226	0.0372±0.0237	1.0080±0.0184	0.7164±0.0123	0.7087±0.0139
**50**	0.0514±0.0210	0.0456±0.0244	0.0381±0.0241	1.1998±0.0271	0.8279±0.0126	0.9467±0.0100

Table 4 Execution time study performed for CNN1, CNN2 and CNN3 using batch sizes from 1 to 50 and two hardware platforms: only an Intel i7 CPU, and adding a GeForce 1080Ti GPU. The values of BS used for the training of each network are marked in blue.

**Class**	**Categories**	**Train**	**Validation**	**Test**
NEG	Normal	105	15	30
POS	LSIL, HSIL & Carcinoma	105 (35x3)	15 (5x3)	30 (10x3)

Table 5 Bethesda System's categories included for each class and number of samples used for each one for the 2-class system.

Batch size	Learning Rate					
	1e-5		1e-4		1e-3	
	Acc	Loss	Acc	Loss	Acc	Loss
**16**	63.04	0.71	98.43	0.09	76.89	0.73
**20**	91.75	0.38	100	0.02	96.67	0.09
**24**	65.60	0.57	97.92	0.10	93.12	0.31
**28**	71.62	0.65	97.99	0.08	97.15	0.11
**32**	80.46	0.67	99.17	0.04	98.33	0.03

Table 6 Test subset results for coarse-grained study with 3 learning rates and 5 batch sizes for the 2-class classifier.

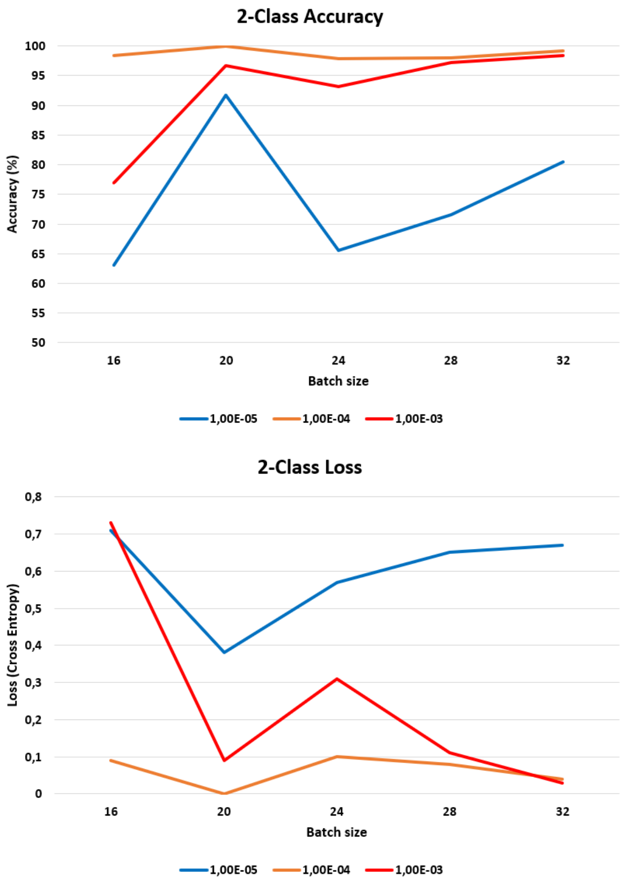

Fig. 4 Accuracy and Loss summary for the 2-class architectures.

Class	Accuracy	Specificity	Precision	Sensitivity	F1$_{score}$
**NEG**	100	100	100	100	100
**POS**	100	100	100	100	100
**TOTAL**	100	100	100	100	100

Table 7 2-class winner architecture final results.

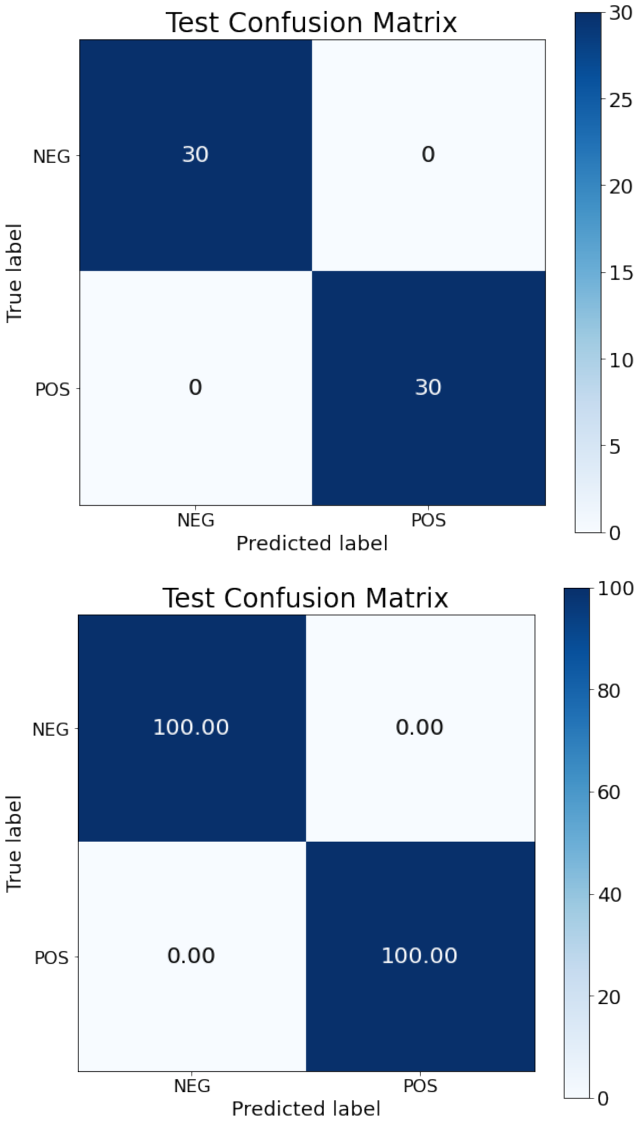

Fig. 5 Test Confusion Matrix for the 2-class winner classifier: (top) Absolute values; (bottom) Percentage values.

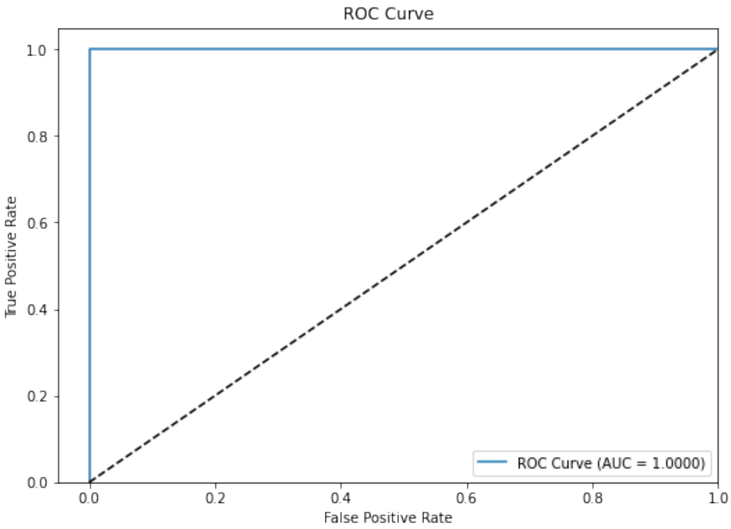

Fig. 6 ROC curve for the 2-class classifier (with AUC value).
